# Design and development of a novel multirotor configuration with counter-rotating coaxial propellers

**DOI:** 10.1038/s41598-024-62289-4

**Published:** 2024-05-21

**Authors:** Mohammad Malakouti Khah, Sayyed Majid Esmailifar, Sepehr Saadat

**Affiliations:** Hardware In The Loop Laboratory, Aerospace Department of Amirkabir University of Technology, Tehran, Iran

**Keywords:** Aerospace engineering, Mechanical engineering

## Abstract

In this study, a novel multirotor configuration has been developed, which utilizes a coaxial counter-rotating propeller with a large diameter at the center and four propellers with smaller diameters on four arms in an X configuration. The research pursues two main objectives. The first objective is to establish a development method for multirotors based on the well-known V-method approach in system development. The proposed method enables the design team to analyse and evaluate the multirotor at each stage of the design process, from the system to the component level, and improve the design with greater speed and accuracy. Additionally, the proposed approach can be integrated with various optimization methods, if necessary, to achieve an optimal design. The second objective is to provide the proof-of-concept of the novel multirotor configuration. This objective is pursued across different evaluation sections, from evaluating individual components to fully evaluating the multirotor, to assess the performance of this novel configuration and identify its advantages and disadvantages. The results of various evaluations demonstrate the proposed development process's practicality and proves that the novel configuration is operational and competitive with conventional multirotors in various applications.

## Introduction

Nowadays, multirotors have experienced significant growth in various applications such as aerial photography, cargo transportation, monitoring, and surveillance due to advantages such as ease of design and development, flight and piloting, vertical takeoff and landing capabilities, hovering in a fixed position, etc. Despite the mentioned advantages, multirotors have various disadvantages, among which the most significant ones include limited flight endurance, range, and payload capacity. Thus, in recent years, scholars and experts have conducted substantial research to address these limitations and enhance the performance of multirotors. These endeavors can be broadly divided into the following three categories:

- Using optimization methods in the design of the multirotor and its subsystems and components: The use of optimization methods in design to select the most suitable combinations of propellers, motors, and other components^1,2^, aerodynamic optimization of multirotors, especially the propellers^3^, structural optimization and weight reduction of the structure^4^, are some of the areas that have been explored to improve the performance of multirotors.
- Utilization of fuel power systems and hybrid power supply systems^5^.
- Development of novel configurations: Exploration of new arrangements, such as using larger propellers to generate the majority of the lift force^6,7^, and the integration of fixed wings alongside rotors^8^, etc.

Numerous studies on rotor and propeller performance have widely acknowledged that increasing propeller diameter, thereby reducing disk loading, significantly enhances propeller efficiency. This efficiency boost directly correlates with increased flight endurance and payload capacity^9–13^. Consequently, this principle has been fundamental in developing more efficient multirotors to improve flight endurance, range, and payload capacity.

**Figure 1:**
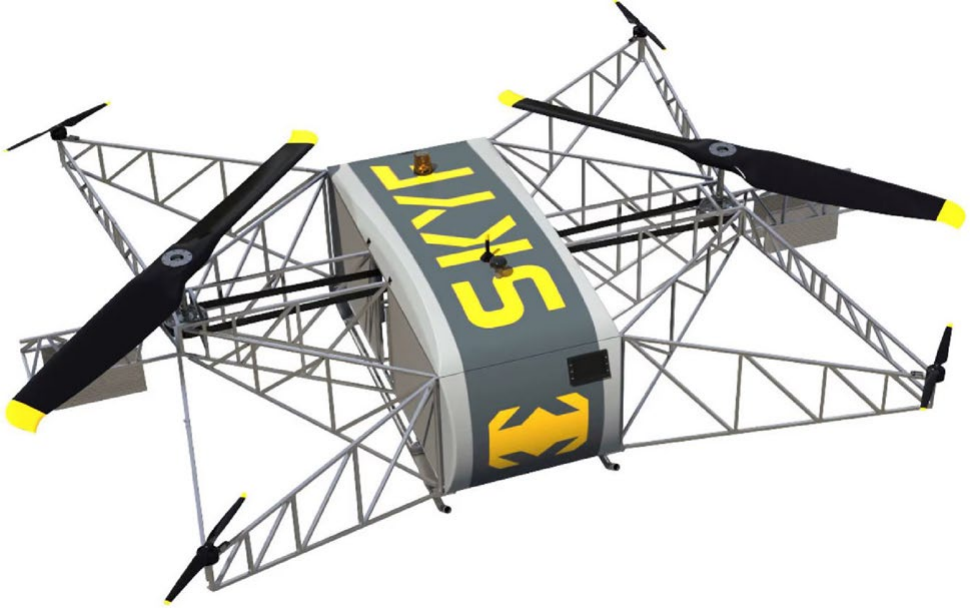
SKYF hybrid multirotor^15^.

In^14^ the design of a long-range hybrid multirotor with high payload capacity was carried out. This aircraft employs two large propellers connected to a fuel-powered motor to generate most of the lift force, while four smaller electric propellers are utilized for its attitude control. The designed multirotor can carry 800 kg of payload with a maximum flight range of 400 km. Like this configuration, ARDN Technology has developed an industrial hybrid multirotor, employing two large propellers to generate lift force, achieving remarkable performance. The SKYF boasts a maximum payload capacity of 181 kg, along with flight endurance and range, reaching up to 8 h and 350 km, respectively^15^. The SKYF hybrid multirotor is shown in Fig. 1.

Using two large propellers with a significant disc area results in higher efficiency of the multirotor in producing lift. However, these large propellers also have some drawbacks, such as requiring more space for takeoff and landing, more storage space, larger takeoff and landing area, and increasing the moment of inertia, which reduces maneuverability. One approach to address these drawbacks is to install the propellers in a coaxial configuration. Coaxial configurations have attracted significant attention in helicopter development due to advantages such as eliminating the need for an anti-torque system, reducing the dimensions of the helicopter, and achieving better flight stability^16^. Additionally, coaxial configurations have been of interest to both industry and academia in many multirotor configurations due to benefits such as dimension reduction and improved resistance to crosswinds^13,17^. While utilizing a coaxial configuration offers advantages, it also presents certain disadvantages, notably mechanical complexity. However, the primary drawback of this arrangement is the loss of aerodynamic efficiency due to the installation of propellers on the same axis of rotation^13,16,17^. Various studies have been conducted to mitigate these aerodynamic losses and enhance the efficiency of coaxial propellers, aiming to make them competitive with single-isolated propeller configurations. The results of these studies show promising advancements in reducing aerodynamic inefficiencies^13,18–20^.

**Figure 2:**
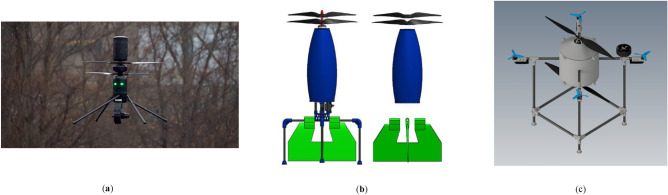
Innovative coaxial configurations: (**a**) SPIRIT^21^; (**b**) developed configuration in^6^; (**c**) novel configuration of the current study.

Based on what was mentioned in the current study, the development of a novel configuration, which utilizes two large propellers in a coaxial configuration and four small propellers to control the multirotor attitude, was carried out. Implementing two large propellers, similar to those used in the SKYF hybrid multirotor^15^ and the hybrid multirotor developed in^14^, could enhance the efficiency of the multirotor. However, adopting a coaxial configuration for these propellers would introduce both the advantages and disadvantages outlined in the previous paragraph to the overall configuration. So, the main purpose of the current study is to provide a proof-of-concept for this novel configuration. The proof-of-concept involves evaluating the feasibility of the configuration and the designed multirotor through various analyses from different perspectives, assessing its advantages and disadvantages. The novel configuration of the current study is presented in Fig. 2c.

A few studies have explored the development of multirotors featuring two large propellers in a coaxial configuration^6,7^. In^6^, the aircraft, which is illustrated in Fig. 2b, employs a pair of coaxial propellers to generate lift force and control the yaw axis, while the control of the roll and pitch axes was achieved using two aerodynamic surfaces positioned beneath the main propellers. The primary focus of the study was to analyze the impact of the coaxial system on these aerodynamic surfaces using CFD tools, with limited analysis provided on the aerodynamic behavior of the coaxial system itself. The authors used some assumptions to provide a simple model for the aerodynamic forces of the coaxial system. Furthermore, the study only conducted ground tests on the multirotor, aimed at verifying CFD results and assessing the effects of coaxial propellers on aerodynamic surfaces.

In study^7^ the method for controlling lift and the yaw axis mirrors that of study 6; however, the aircraft in this study employs a swashplate mechanism to manage the roll and pitch axes. The primary objective of the study was to develop a controller algorithm using sliding mode PID, with less emphasis on the design and development process. While utilizing the swashplate mechanism eliminates the need for additional rotors or actuators to control the multirotor, it introduces significant mechanical complexity to the system. This complexity can lead to increased maintenance issues and higher production costs for the final product.

Although the configuration developed in the current study differs from the ones developed in^6^ and^7^ due to the usage of small propellers to control the multirotor attitude, it shares certain advantages with the configurations developed in those studies. In^6^, the “high-speed cruise” mode was mentioned as one of the most remarkable features of the configuration.

ASCENT AEROSYSTEM^21^ has developed a coaxial configuration named Spirit, which is illustrated in Fig. 2a. SPIRIT has a similar configuration to the one mentioned in reference^7^. SPIRIT exhibits significant performance advantages compared to conventional configurations. Notably, two key characteristics of Spirit's performance, which can relate to the novel configuration of current research, are wind resistance (i.e., forward flight speed) and endurance. Table 1 presents a comparison of these performance characteristics for Spirit and several other conventional configurations.

**Table 1:** SPIRIT multirotor key performance characteristic compare to several other conventional configurations.

Name	Maximum takeoff weight (kg)	Flight endurance	Payload (kg)	Wind resistance (km/h)	References
SPIRIT (coaxial with swashplates)	6.1	53 min with no payload 32 min with max payload	3	65	^21^
DJI Inspire 2 (conventional quadrotor)	4.2	27 min with payload	0.25	36	^22^
DJI MATRIX 600PRO (conventional hexarotor)	15	38 min with no payload 18 min with payload	5.5	29	^22^
Autel Alpha (conventional quadrotor)	8.4	40 with no payload	–	43.5	^23^

As mentioned, the current study presents a proof-of-concept for a novel configuration to aid future researchers in creating more optimized electrical and hybrid prototypes. A systematic and integrated development process is essential for this purpose. In contrast to fixed-wing aircraft, particularly manned airplanes (e.g., reference^24^), the literature reveals a notable absence of a comprehensive design and development process that incorporates all aspects of multirotors and the various disciplines involved in the development process and their interrelations.

Some studies, including^14,25^, directly select and design different subsystems in a specific order and avoid complex relationships. Design teams choose subsystems based on requirements, cost, available products in the market, etc. The final design is produced after convergence testing, considering parameters such as flight endurance, total weight, and payload weight. While these studies offer practical insights into the design process, they often overlook the diverse disciplines relevant to multirotors, such as aerodynamics, flight dynamics, control, structures, and their interrelation and effect on different subsystems. These methods fail to provide adequate solutions when off-the-shelf products are unavailable, which forces the design team to design and develop subsystems and components from scratch. For instance, as detailed in subsequent sections, the current study necessitated a comprehensive design process for the multirotor's flight control subsystem and airframe. Other studies, such as^6,26,27^, focus solely on one or two specific subsystems or components and the associated discipline. The propulsion subsystem, which typically includes propellers, brushless DC electric (BLDC) motors, electronic speed control (ESC), and batteries, is often the primary focus. While these studies offer optimized approaches for selecting the best combination of propellers and related components, they overlook other subsystems and disciplines. However, as demonstrated in the development process of the current research, these overlooked aspects can significantly impact each other, as well as aerodynamic performance and the designed propulsion subsystem.

Some research, such as^1,2,28^, utilize optimization techniques to design a multirotor that satisfies specific requirements, optimizes various characteristics, and considers different subsystems and their relationships. To achieve the optimization objective, these methods require modeling multiple aspects of the multirotor, such as propeller aerodynamics, brushless motors' electrical characteristics, and weight estimation. Although these studies adequately present relationships between various components and subsystems, they also exhibit shortcomings. For instance, details regarding optimal configuration selection, such as the number of propellers, their arrangement, etc., still need to be elucidated. Moreover, the complexity of different disciplines makes using intricate numerical methods in optimization costly, forcing designers to use computationally simpler approaches or ignore some disciplines. For example, in^1^, for estimating the strength of an airframe, a simplistic model for stress on the arms under maximum thrust conditions is utilized, whereas, the present research demonstrates that the highest stress on the structure occurs during sudden dynamic loads, such as during landing. Also, utilizing simplistic models may make the optimization algorithm's final design and proposed components impractical. For instance, in the method proposed in^1^, there is an approximate 10–15% disparity between the final weight of the multirotor and the estimated weight by the algorithm or between the estimated power consumption and the actual power consumed. These disadvantages could pose difficulties if the intention is to implement and build the multirotor physically.

Based on the previously mentioned research, an integrated and systematic approach was established for developing the multirotor in this study. This method utilizes the V-method. The V-method organizes all stages of the multirotor’s design and development, from the initial requirements analysis to the overall system design, the design of subsystems, and the detailed design of components and their interconnections within a structured framework. It also considers the physical development stages, the evaluation of different system parts, and the interactions between various disciplines. This approach enables the design team to benefit from the pragmatic advantages mentioned in^14,25^ while maintaining a holistic systems perspective and the interrelation of components and disciplines in line with achieving an optimal design, as mentioned in^1,2,28^. To the best of the author’s recollection, this research represents the first application of this method in developing a novel multirotor with the aim of considering all subsystems and developing a laboratory prototype.

Due to the multidisciplinary nature of the development process, numerous studies have explored various aspects and disciplines related to multirotors. These studies are discussed in the following paragraphs.

During the aerodynamic analysis of the multirotor, the most important aspect is the analysis of the propellers, particularly coaxial propellers. In^6^, a simple model is used to calculate propellers' aerodynamic forces and moments. In^7^, a combination of blade element momentum theory (BEMT) and dynamic inflow model is utilized to simulate the behavior of coaxial propellers. Researchers widely utilize the BEMT to analyze rotors due to its simplicity, low computational cost, and suitable accuracy for early design stages^18,29^. Numerical simulation using Computational Fluid Dynamics (CFD) can be conducted to analyze coaxial rotors further. CFD simulations for multirotor propellers have been carried out in various references, such as^30,31^.

According to^32^, in most studies, the drag of small multirotors is considered negligible, and its effects on the multirotor’s flight dynamics are not considered. Bangura^33^ extensively examined the drag of multirotors and provided a helpful classification. In some references, such as^25,34^, it is suggested to calculate the drag from flight test results and accelerometer outputs. CFD simulations for estimating the drag have also been conducted in some references, such as^35,36^.

Extensive research and studies are available for multirotor flight dynamic modeling, simulation, and flight control system development. In^7^, a 6-degree of freedom (DOF) modeling is established for the coaxial configuration, and a combined PID and sliding mode controller is designed and compared with a conventional PID controller in terms of performance. Similarly, in reference^6^, modeling and simulation are conducted for the multirotor, and a controller is designed using the pole placement method. Reference^37^ presents a 6DOF modeling of a multirotor and a controller design based on the PX4^38^ autopilot. In the autopilot development for specific applications (e.g., new configurations), research studies have considered different approaches for implementing autopilot systems on a multirotor; there are three main solutions:

- In^39^, the development of an autopilot system using an Arduino Mega 2560 microcontroller is presented. It successfully performs autonomous flight with a mission to follow a desired path for a DJI-S800 multirotor. Similarly, reference^40^ implements a multi-module system for controlling a quadrotor, with the autopilot implemented on a Raspberry Pi microcontroller.
- Using a microcontroller or companion computer alongside the PX4 autopilot is another solution for enhancing and developing the autopilot's performance for a specific application. For instance, in^41^, an Odroid U3 OBC companion computer is used with the Pixhawk autopilot board, serving as a ground station and sending necessary commands such as changing flight modes, etc.
- Many other studies have also included using commercial autopilots such as PX4 and making necessary modifications if required. In^37,42^, the development of a MATLAB toolbox for implementing and modifying the PX4 autopilot firmware, which is then used to build the final firmware on target autopilot boards, such as Pixhawk 1, is presented. Other references, such as^43,44^, directly modify the PX4 autopilot code for their specific usages. Reference^43^ changes the control architecture of PX4, replacing the existing control logic with a retrospective cost adaptive control (RCAC), and evaluates its behavior under conditions of uncertainty in the moments of inertia of a quadrotor. Similarly, reference ^44^ develops a new hybrid aerial robot configuration and develops the necessary control and guidance logic for the PX4 autopilot. Finally, software-in-the-loop (SITL) testing is performed by modeling the aircraft in the Gazebo environment.

Incorporating commercial autopilot controller architecture into the flight dynamic simulation stage presents notable benefits for design teams. By integrating these systems, designers can harness the capabilities of commercial flight control subsystems throughout the development process. This approach streamlines the utilization of well-established technology, facilitating efficient and cost-effective implementation.

In the structural analysis of multirotors, reference^45^ conducts simulations of various static and dynamic loading scenarios on a quadrotor using the Ansys Mechanical software and examines the results. Similarly, in reference^4^, the structural optimization of a quadrotor is performed by simulating different configurations. Existing studies focus solely on the weight of the multirotor and propeller thrust force as primary loading factors on the airframe. However, the analysis in the current research emphasizes that dynamic loading during “hard landing” scenarios is the most critical consideration.

Table 2 illustrates a summary of what mentioned above to clarify the purposes of the study and its contribution.

**Table 2:** Summary of the advantages and disadvantages of previous work and the contribution of the current study.

Innovation	Purpose	Similar researches or industrial products	Advantages	Disadvantages	Contribution of the current study
Proof-of-concept for a novel configuration with large coaxial propeller in center to generate the majority of the lift force and four control propellers in quadrotor configuration for attitude control (See Fig. 2c)	Future hybrid multirotors (an improvement to multirotors such as SKYF^15^)	^14,15^ (See Fig. 1)	Improved flight endurance, range and payload capacity	Increased moment of inertia which reduces the maneuverability Increased storage space, takeoff and landing area	Utilizing propellers in a coaxial configuration can help overcome the mentioned shortcomings
	Future electric multirotors	^6^ (See Fig. 2b)	Utilizing coaxial configuration has improved the forward flight speed CFD analysis carried out to measure the effect of the propellers on aerodynamic surfaces	Proof-of-concept was provided by ground test to verify CFD results Less focus on aerodynamic behavior of propellers in flight dynamic simulation	Attitude control is provided by using four control propellers in quadrotor configuration Proof-of-concept was provided through more detailed evaluation and flight tests Aerodynamic model of propellers used in controller design section is based on blade element momentum theory and CFD corrections
		^7,21^ (See Fig. 2 (a))	Utilizing coaxial propellers with swashplate mechanism Excellent performance in flight endurance and wind resistance	Swashplate mechanism adds mechanical complexity to the system which can lead to increased maintenance issues and higher production costs Lack of a thorough design and development process	Attitude control is provided by using four control propellers in quadrotor configuration A Systematic procedure for design and development process based on V-method is introduced
Establish a systematic and integrated design and development procedure based on V-method	To provide a systematic approach for practical development, encompassing all system levels and disciplines involved	^14,25^	Practical perspective of design process which leads to fast development	These studies overlook diverse disciplines crucial to multirotors and their interrelation and impact on subsystems These methods struggle when off-the-shelf products are unavailable, necessitating the development team to create systems from scratch	Current study considers different disciplines and their effect on different subsystems and components Provides different validation steps to validate the multirotor system in different level of developments
		^6,26,27^	Effectively optimize different component of the multirotor	Their primary focus is solely on one or two specifics subsystems or components (usually propulsion) and the associated discipline	Current development process considers all of the subsystems and associated discipline
		^1,2,28^	Utilizing optimization methods to select the best combination of subsystems and components Provide a good understanding of the multirotor system architecture	Optimization methods could not be used for optimal configuration selection, leading to ambiguity in design decisions at the early stages of design Complex analyses method cannot be used, forcing the design team to ignore some disciplines or utilizing simpler methods with less accuracy Utilizing simpler analyses tools will lead to disparities between estimated and actual design variables	This approach enables the design team to benefit from the pragmatic advantages mentioned in^14,25^ while maintaining a holistic systems perspective and the interrelation of components and disciplines in line with achieving an optimal design like what mentioned in^1,2,28^

### Overall design and development procedure

**Figure 3:**
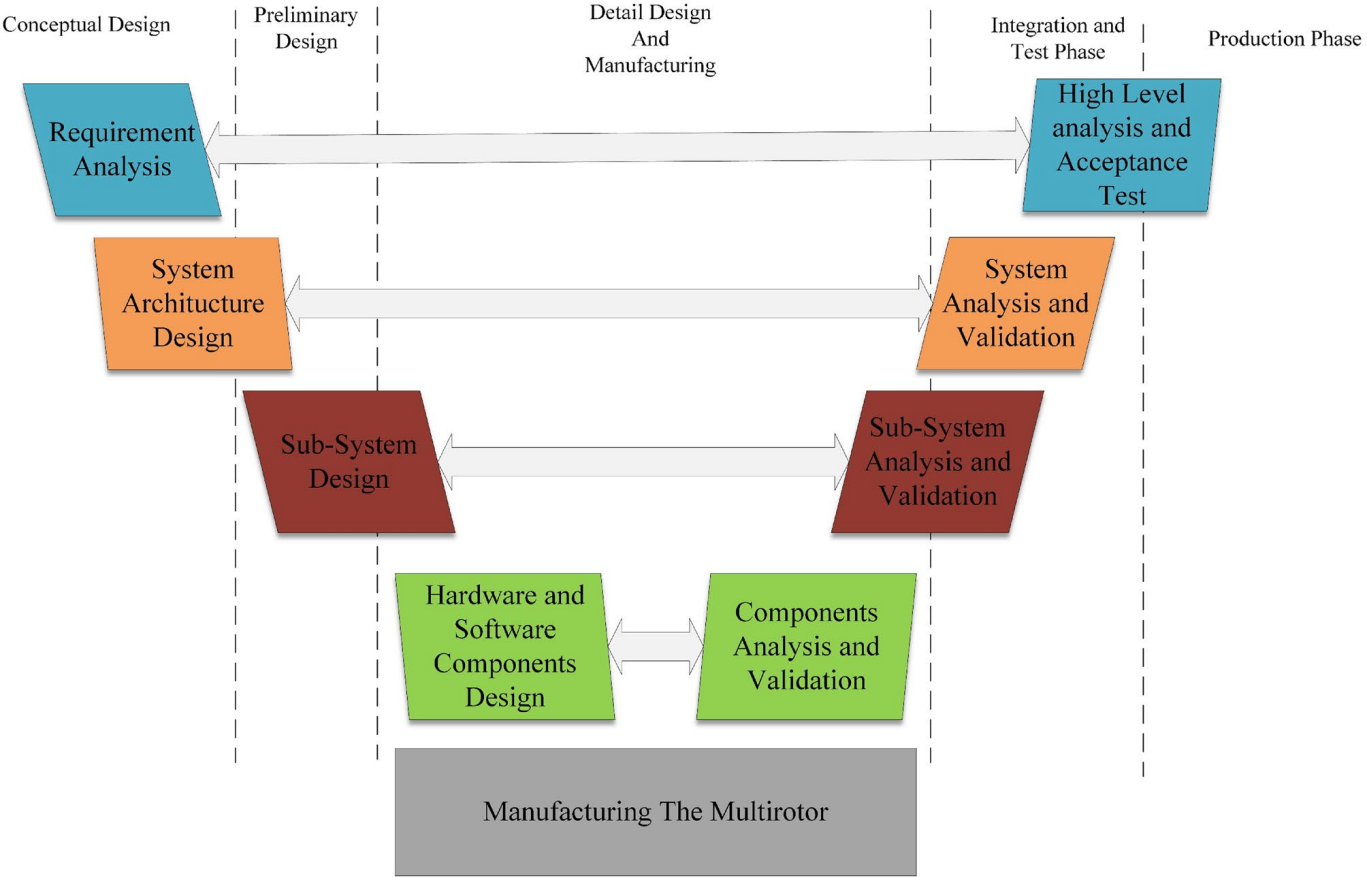
The overall design and development process.

This article’s overall design and development procedure is based on a V-Diagram illustrated in Fig. 3.

The first stage calculates the requirements such as performance characteristics, weight and size specifications, cost, maintainability, handling qualities, etc. These requirements can be dictated by customers, market analysis, research and development criteria, etc.

In the second step, *system architecture design*, characteristics such as configuration, placement, and overall specifications of different subsystems and their components will be determined. Also, the development process for each subsystem should be decided in this step. The development process for various subsystems can differ based on requirements; for example, in the current study, the airframe and flight control subsystems require a vast series of designs and development, which should be carried out in the “*hardware and software component design*” phase. Still, on the other side, the power supply subsystem components do not need further design beyond what is carried out in the “*subsystem design”* phase, and their development only includes purchasing the components and some soldering to prepare the components for assembly and integration.

After determining the overall characteristics, each subsystem's overall parameters, specifications, and requirements will be imported into the *subsystem design* phase. The design of subsystems includes determining the necessary parameters for each subsystem to satisfy all requirements. Here, we employ the method in^14,25^, which considers a design loop in which different subsystems are designed in a specific order, and finally, the convergence of the design loop will be examined.

In the *hardware and software component design*, based on the development process determined in the *system architecture design* phase, different components are designed and developed for each subsystem.

In the validation part of the V-method on the right side of Fig. 3, a wide range of analysis could be carried out to evaluate the performance of components, subsystems, and the multirotor system. Theses analysis and their result are presented in following sections:

- In *components analysis and validation*, different components from different subsystems can be analyzed. In the current study, the most critical component that will be discussed in this stage is the software and autopilot board of the *flight controller system*.
- In *subsystem analysis and validation*, the multirotor subsystems are analyzed based on aspects such as aerodynamics, flight dynamics, and structure. Each analysis involves different subsystems; for example, the structure analysis involves mainly the *airframe subsystem* and considers the constraints and conditions from other subsystems, and flight dynamic analysis consists of a combination of the *flight control subsystem*, *aerodynamic force and moment generation subsystem*, etc.
- The *system analysis and validation* of the multirotor system is about conducting flight tests. This process may be lengthy and could begin with simple ground tests to ensure that the system and its subsystems are functioning correctly and extend to include flight tests for measuring performance characteristics and identifying multirotor parameters.

At the final stage of the development process, high-level validation is essential to evaluate if the multirotor can satisfy the necessary (high-level) requirements. These validations can include various evaluations, including flight tests, structural strength tests, ease of assembly and maintenance evaluation, etc. Because the developed multirotor is for proof-of-concept, the evaluation of high-level requirements doesn't need to be covered in this article and is postponed to future studies.

Although this design and development process differs from conventional procedures like what is addressed in^46^, which is mainly for manned fixed-wing airplanes, some correspondences could be found between conventional design steps such as conceptual design, preliminary design, etc., and the method illustrated in this article. This is also shown in Fig. 3.

## Requirement analysis

As mentioned before, the multirotor is developed for proof-of-concept; therefore, most requirements are based on research targets considering cost and ease of development.

- The multirotor has a novel and non-conventional configuration. Therefore, in the first step, the proof-of-concept for this new configuration should be considered, i.e., it should be examined if this configuration can be developed and also if the developed multirotor can perform as a normal multirotor and executes the maneuvers and missions that are expected from a conventional multirotor. Also, the investigation of the advantages and disadvantages of this multirotor is necessary and should be carried out during the design, development, and validation process.
- The multirotor should employ a pair of counter-rotating coaxial propellers, which (considering the future development purposes for hybrid configurations) should generate the majority of the lift force. The number of control motors is arbitrary and can be decided in the design process.
- The development process of the multirotor should be inexpensive and straightforward, with no need for complex machining and tooling.
- The multirotor should be stable and controllable with no need for configuration modification (for example, using another actuator to stabilize and control the multirotor). The handling quality should be at the same level as an electrical multirotor with conventional configurations with similar size and weight characteristics.
- The multirotor should be able to carry a payload. The multirotor payload was considered a box with a 10 × 10 × 5 cm dimension. Considering the large coaxial propellers, this requirement is essential and can challenge the novel configuration and its performance compared to conventional configurations. Table 3 contains the payload weight requirement of the multirotor.
- The multirotor hover endurance should be comparable with an electrical multirotor with conventional configurations with similar size and weight characteristics.

**Table 3:** Payload and hover endurance requirement.

Requirement	Value
Payload weight (g)	200
Hover endurance (min)	15

## System architecture design

**Figure 4:**
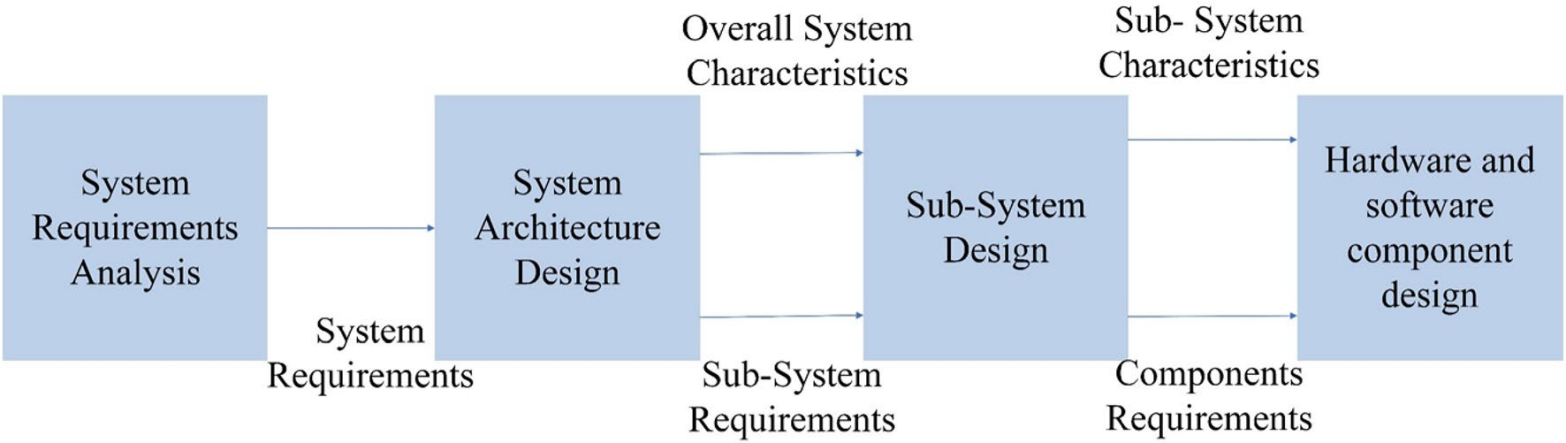
Characteristics and requirements hierarchy.

In this section, the multirotor is first divided into different subsystems; then, the requirements from section “Requirement analysis” are considered and translated into more detailed requirements for each subsystem. The exact process will be carried out for *the hardware and software component design* stage, as illustrated in Fig. 4.

Also, the overall characteristics of the multirotor system will be calculated, and the development process for each subsystem will be determined. Finally, the placement of subsystems and the multirotor configuration will be decided.

**Figure 5:**
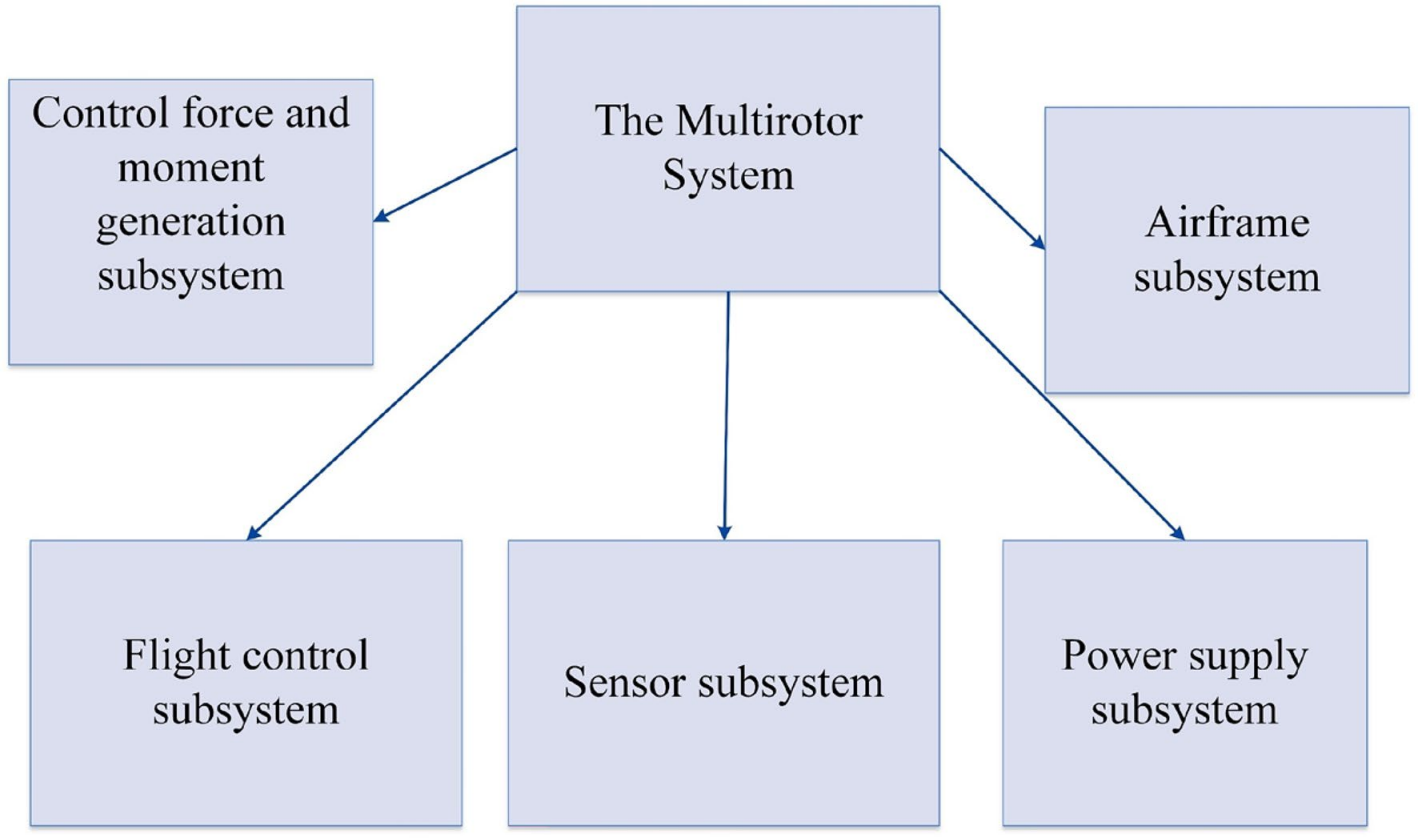
The multirotor subsystems.

A Multirotor can be divided into different subsystems from different aspects^14,25^. Here, we divide the multirotor into five subsystems, illustrated in Fig. 5.

- *Control force and moment generation subsystem*: This subsystem includes propellers, BLDC motors, and ESCs. In the current study we divide this subsystem into two parts: main coaxial propellers, their BLDC motors and ESCs, and control propellers, their BLDC motors and ESCs.
- *Flight control subsystem*: The autopilot is the primary component of the flight control subsystem. Like the human brain, the autopilot stabilizes, guides, and controls the multirotor to complete its mission successfully. To accomplish this task, the autopilot receives information about its current state from various sensors inside and outside the autopilot board, such as IMU, GPS, barometer, etc. Subsequently, using implemented guidance algorithms, the multirotor's current state is compared to the desired state to complete the mission, and the necessary commands are applied. These commands are usually in the form of PWM signals that determine the rotation speed of different motors by being transmitted to the ESCs.
- *Sensor subsystem*: In the current study, the sensor subsystem includes sensors that are not part of *the flight control subsystem* but are necessary for the multirotor to complete its mission, for example, different cameras, LIDAR sensors, etc.
- *Power supply subsystem*: This subsystem comprises a battery, a power distribution board, and a power module. In most electric multirotors, LiPo batteries are used due to their high energy density in smaller dimensions and weight and their high voltage per battery cell compared to other batteries such as lithium-ion, etc. These batteries are typically connected to a power distribution board, which distributes electrical power to the brushless motors, autopilot board, and some sensors. In some multirotors, a power module transmits the required power to the flight control system.
- *Airframe subsystem*: Airframe subsystem is the structure that holds all the other subsystems together in a specific configuration.

### Determination of overall characteristics, requirements, and development process for subsystems

The requirements in section “Requirement analysis”, can be translated into more detailed requirements for each subsystem and also be utilized to calculate overall characteristics and development process for each subsystem.

- Control force and moment generation subsystem: A pair of counter-rotating coaxial propellers should be employed as a main thruster of the multirotor. To control the multirotor, at least three control propellers should be utilized. This step should calculate two main parameters: the thrust ratio of the main propellers to control propellers, and the number of control propellers. An initial guess of the multirotor maximum take of weight ($${W}_{TO})$$ is necessary for an acceptable calculation of these parameters. This initial guess can be made using the data from multirotors with a similar configuration, weight class, etc. Still, due to the novel configuration of the multirotor, there is a shortage of statistically significant data available on multirotors with comparable configurations. Therefore, our analysis is focused solely on similar multirotors concerning weight. Specifically, we examined ten multirotors with a maximum takeoff weight of less than 4 kg, and their information is presented in Table 4.

**Table 4:** Specifications of some available multirotors in the market.

Manufacturer	Model	Payload weight (g)	Hover endurance (min)	${\varvec{W}}_{{{\varvec{TO}}}}$(g)	References
DJI	PHANTOM 4PRO	290	28	1388	^22^
Height Technologies	Marlet MI1	200	40	1600	^47^
Yuneec	Typhoon H3	567	25	1985	^48^
Autel Robotics	EVO II	500	35	1999	^23^
Yuneec	H520E	300	30	2186	^48^
Swellpro	Splashdrone 3 +	1000	20	3000	^49^
Swellpro	FisherMan	1000	15	3050	^49^
DJI	Metrice 100	500	16	2431	^22^
UAV Systems	Tarot 650 v2.2	1500	15	3550	^50^
Height Technologies	Marlet MI2	1000	55	4000	^47^

A graph was generated using available data and employing a curve fitting tool to calculate the correlation between payload weight, hover endurance, and maximum takeoff weight.

The equation that represents the correlation is:

1 $$W_{T.O} = 244 + 2.07W_{P} + 31.27 t_{hover} .$$

In the above equation, ${\text{W}}_{{{\text{TO}}}}$ and ${\text{W}}_{{\text{P}}}$ are in grams, and ${\text{t}}_{{{\text{hover}}}}$ is hover time in minutes. The coefficient of determination (R^2^) for this correlation is 0.83, indicating the suitability of the equation for initial weight estimation. Using the above equation and inputting the values of flight endurance and payload weight from Table 3, the maximum takeoff weight of the multirotor was calculated as 1200 g.

The more detailed design of this subsystem is part of *the subsystem design* phase. Still, at this stage, an appropriate decision could be made considering the performance test results of different propellers on the market published by manufacturers like Tmotor^51^. The result test data are accurate for control propellers installed as single isolated propellers, but for coaxial propellers, using the result of single propellers is not a proper method. In *the subsystem design* phase, the BEMT method will be utilized to analyze the performance of coaxial propellers; based on this method, a 20%-30% drop in total thrust of a coaxial system compared to two separate propeller could be an appropriate raw guess to use single propeller test results for the coaxial system. Using the method mentioned above, considering the criteria and based on^25,52^, the following results were concluded:

1. The ratio of coaxial propellers' thrust force to control propellers' thrust force is calculated as:
  2 $$\frac{{T_{Coaxial} }}{{T_{control} }} = \frac{0.8}{{0.2}} = 4 .$$
2. In hover (i.e., throttle about 50% to 60%), the propellers should generate thrust force equal to $W_{TO} .$
3. At maximum throttle, the propeller's thrust should be twice the value of $W_{TO} .$
4. Four propellers in the configuration of X quadrotor should be utilized as control propellers; using more motors, like six or eight, will make the thrust share of each control propeller so small that it would be difficult to find an appropriate propeller and BLDC motor in the following development stages.

Considering the calculated $W_{TO}$, assumed thrust ratio and mentioned requirements, the thrust for coaxial and control propellers can be determined as mentioned in Table 5.

**Table 5:** Specifications of some available multirotors in the market.

Part	Hover thrust (N)	Maximum throttle thrust (N)
Coaxial propellers	9.7	18.8
Control propellers	2.4	4.7

Many commercial and industrial multirotor manufacturers design and build their required propellers according to design needs and performance optimizations. Still, since the development process should be inexpensive with no need for tooling and complex manufacturing processes, the development of this subsystem only includes purchasing the selected propeller, BLDC motors, and ESCs and preparing them to assemble with other parts and subsystems.

- Flight control subsystem: At this stage the most important decision for this subsystem is the development process. As mentioned in section “Introduction”, different methods exist to develop a new autopilot. Following an investigation of various methods, it was decided to utilize the PX4 autopilot firmware and develop it further to incorporate the new configuration of the multirotor. The use of PX4 autopilot firmware presents several advantages. Firstly, the firmware supports using standard autopilot boards and sensors that are compatible with PX4, eliminating the need for custom hardware development, which can be both time-consuming and expensive. Secondly, integrating the novel configuration into the PX4 autopilot firmware allows the multirotor to leverage the various submodules available within the PX4 autopilot system, such as different flight modes and compatibility with other sensors for various mission requirements.
- Sensor subsystem: As mentioned in section “Requirement analysis”, the multirotor is for proof-of-concept purposes, and no specific mission is considered. Therefore, there is no need for sensors beyond the flight control subsystem sensors. Therefore, there is no design and development process considered for this subsystem.
- Power supply subsystem: The most important component in this subsystem is the battery. As mentioned, LiPo batteries are the most common power source in electric multirotors. Therefore, a LiPo battery will be utilized. The requirement for the LiPo battery can be summarized as below:
  1. Compatible voltage (i.e., number of cells) with ESCs and motors: Each LiPo battery cell provides 3.7 V as nominal voltage to 4.2 V as maximum voltage. Therefore, the number of series cells in a LiPo battery is essential.
  2. Sufficient energy capacity to fulfill endurance requirements: The multirotor ${\text{W}}_{\text{TO}}$, along with hover flight endurance, calculates the Ah capacity of the LiPo battery. The LiPo Battery should have an Ah of at least 120–125% of the Ah which BLDC motors need to hover at maximum ${\text{W}}_{{{\text{TO}}}}$ with endurance mentioned in Table 3.
  3. Maximum discharge rate: This parameter of the LiPo battery should be high enough that all motors can draw their maximum required current without damaging the battery.

For other components of the power supply subsystem, like the power distribution board (and power module), the requirements are combability with the battery and supporting enough outputs (for the power distribution board) for all ESCs. The development process for this subsystem includes only purchasing the components and preparing them to assemble with other subsystems and components.

- Airframe subsystem: The requirements for the airframe subsystem are:
  1. Being inexpensive
  2. Ease of assembly and build with no need for complex machining and tooling
  3. Ease of maintenance in case of a crash or damage
  4. Ease of access to different components based on their usage: For example, the user should be able to connect and disconnect the ESCs to BLDC motors, battery to the power distribution board, battery to the charger, etc., with no need to disassemble the multirotor.

Due to the novel configuration of the multirotor, it is not feasible to use the available airframes in the market. Therefore, a complete design and build procedure must be considered. Based on this development process and the mentioned requirements, it was decided that the multirotor should use carbon-epoxy composite plates and tubes as the primary material. Also, the 3D-printed parts could be used for connections if no appropriate connection is available in the market.

### Overall configuration of the multirotor

At the final step of the system architecture design, the configuration of the multirotor and the placement of different parts and components should be considered. Initially, various configurations, should be considered. Subsequently, each design option should be evaluated from various perspectives. Finally, the most suitable design should be selected while considering different criteria. Choosing the best choice among multiple options can be challenging. Therefore, the current design process utilizes a simple version of concept scoring selection method^53,54^ to identify the proper design.

**Figure 6:**
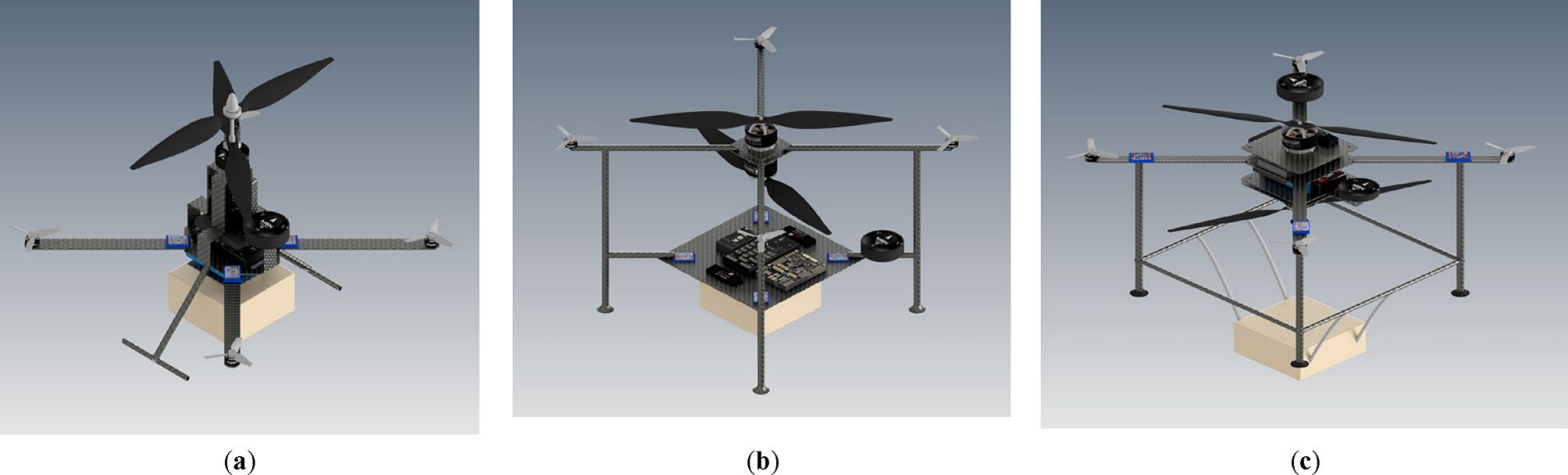
Three options for the multirotor configuration: (**a**) Design 1; (**b**) Design 2, (**c**) Design 3.

After investigating different options for shapes and arrangements, three designs were selected for further analysis, illustrated in Fig. 6.

The first design differed from the other two by co-axially assembling the motors with a large shared shaft instead of simply installing them on separate plates. The second and third designs had two motors installed on separate plates without a mechanical connection. In the second design, electronic components were placed on a large plate at a suitable distance from the blades, while in the first and second designs, they were installed in plates under and between the main blades, respectively. These designs were examined considering the following aspects.

- Coaxial propellers arrangement: Reducing the distance between blades can increase disruptive effects and violate assumptions of the BEMT, and increasing the distance will increase the design weight and dimensions. All designs meet the distance requirements of BEMT (i.e., at least 0.25 of upper propeller radios^29^), but the third design provides more distance, improving blade performance. To optimize aerodynamics, it's ideal to have no blocks between or beneath the blades. If blocks are present, just like the case in the current study, their dimensions should be small relative to the propeller’s diameters, and their distance from the blade surface should increase as their size increases. Placing small components near the blade surface has little effect on their performance, but larger components require more distance^55^. The first and third designs will provide better performance than the second design as they have more free space between blades.
- Ease of Manufacture: Efforts were made in all three designs to maintain simplicity in construction methods and components. The first design has the highest construction complexity due to its mechanism for coaxial propeller rotation. The second design has the most elevated simplicity, with no complex mechanism between the blades and all components placed on one plate.
- Structural Strength: At this stage of the design process, a preliminary and expedited structural analysis must be conducted to estimate crucial design parameters, such as the dimensions of various parts and the structure's weight. Additionally, different designs must be evaluated from a structural strength standpoint. Hard landing is one of the most critical loading conditions on the multirotor structure^14^. The second design has relatively higher strength due to the presence of a main plate in the lower part of the device, which reinforces the landing skids, particularly in the shear direction. The third design also has suitable reinforcement due to connections between the landing skids. The first design has shorter landing skids, which will apply less torque at the connection point, but the angle of the skids causes some of the imposed compressive force to act as shear force and torque. Structural analysis in hard-landing at this design stage can be conducted by a simple quasi-static method like the one mentioned in^14^ and considering the carbon-epoxy composites as an isotropic material^25^.
- Weight: The overall weight of the design is also essential; the subsystem that makes a difference between the weight of different designs is the airframe subsystem. Using the data from multirotors in the same weight class, an acceptable estimation of the dimensions and thickness of part of the airframe can be made, and a reasonable estimate of the airframe weight can be calculated. This estimation can be more accurate by using the result from the structural analysis mentioned above.
- Ease of assembly: Among the mentioned designs, the second design offers advantages, where all components are easily placed on a middle plate. The third design provides greater ease of access than the first design since the components are installed on main plates with simple rectangular shapes.
- Ease of payload placement: Payload placement is challenging due to their impact on coaxial propellers efficiency, overall weight, etc. The presence of a payload under the propellers in the third design creates a blockage effect during takeoff, but this can be controlled by adjusting its dimensions. In the first and second designs, the payload is located under other components and has no effect on propellers. However, an increase in height is required to place the payload, resulting in approximately 7% increase in height for the first and second designs when they want to carry the payload. Therefore, there is no significant advantage among the designs in this aspect.
- Esthetic Appearance: While the primary objective of this project is to demonstrate proof-of-concept, the multirotor design's appearance can significantly impact its further development. A visually appealing and distinctive configuration can positively influence potential investors. The second design is unappealing among the proposed designs due to its long skids, separation of middle motors, and placement of most electronic components on one plate. The first design's complex shape make it difficult to design add a cover to the multirotor which can significantly improve the appearance. The third design, with a simple cylindrical cover, offers a more suitable appearance by concealing its complexities and enhancing its aesthetic aspect.

The concept scoring selection method^53,54^ will be considered to select the best design. In this method, all criteria will first be weighted based on relative importance with a summation of weights equal to 1 (or 100%). Next, one of the designs (the first design here) is considered as a reference for rating. Rates would be from 1 to 5, with a higher rate meaning better performance in criteria, and reference design will have a rate of 3 in all measures. After that, other designs will be rated based on analysis and judgments made before. Finally, the score for each design in each criterion will be calculated by multiplying the criteria weight and design rate. The design that obtains the higher score is selected as the suitable design. This process is shown in Table 6.

**Table 6:** Specifications of some available multirotors in the market.

Criteria	Weight	Design (1)		Design (2)		Design (3)	
		Rate	Score	Rate	Score	Rate	Score
Coaxial propellers arrangement	0. 4	2	0. 2	1	0.6	3	20%
Ease of Manufacture	1.05	4.2	1. 25	5	0.75	3	25%
Weight	0.4	2	0. 3	1.5	0.6	3	20%
Ease of assembly	0. 35	3.5	0. 4	4	0.3	3	10%
Ease of payload	0. 15	3	0. 15	3	0.15	3	5%
Structural strength	0. 56	3.75	0. 67	4.5	0.45	3	15%
Esthetic Appearance	0. 19	3.75		0.1		2	
sum		3.1		3.07		3	

Therefore, the third design was selected as the best option.

## Subsystem design

**Figure 7:**
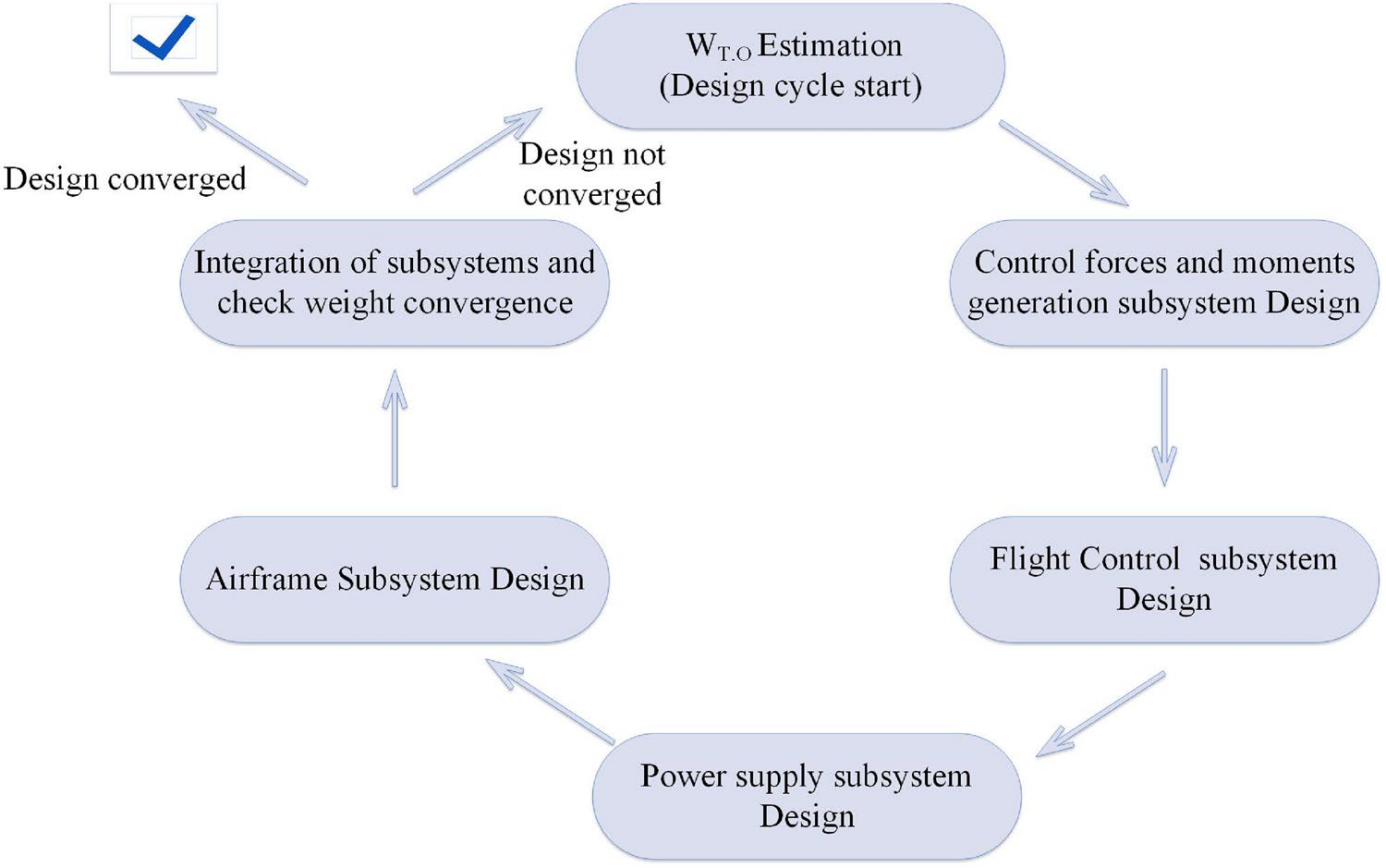
Subsystem design cycle.

In this part, the detailed design of each subsystem will be carried out using the subsystem level requirements and overall characteristics of the multirotor system determined in section “System architecture design”. The subsystem design phase could be complicated, time-consuming, and iterative, with many interconnections between different subsystems. There are various methods in the literature to overcome these challenges, as mentioned in section “Overall Design and development procedure”; we utilize a method similar to^14,25^. In this method, a design cycle, illustrated in Fig. 7, is considered in which each subsystem will be designed in a specific order based on requirements in section “System architecture design”. Finally, the convergence of the design will be evaluated.

This cycle will be initiated based on the initial guess of $W_{TO}$ in section “System architecture design”. The control force and moment generation subsystem will be designed as the first subsystem. After that, the flight control subsystem and sensor subsystem (if applicable) can be designed. The power supply subsystem can be designed in the next step based on the electrical power required for the multirotor, especially the BLDC motors. Finally, the airframe subsystem could be designed. All the requirements in section “System architecture design” will be analyzed, and necessary design parameters will be calculated to satisfy the requirements.

After designing all the subsystems, the weight of the multirotor can be calculated as:

3 $$W_{T.O} = \mathop \sum \limits_{i = 1}^{n} W_{i} + W_{P} .$$

In which (n) is the number of subsystems. Comparing the weight calculated from Eq. (3) to the estimated weight at the beginning of the design cycle, the convergence of the design cycle could be evaluated. If the difference between two weights is less than 5%, the design is considered complete; if not, the re-estimation of ${W}_{T.O}$ would be necessary. (In this article, only the last design cycle is presented.)

### Control forces and moments generation subsystem design

This subsystem's design begins with the propellers' analysis and selection. As mentioned before, there is a shortage of comprehensive data on test results for coaxial propellers. Consequently, it becomes necessary to adopt a methodology to determine their performance specifications and characteristics. Various methods are available for analyzing coaxial propellers. However, in this stage, due to the broad range of available options, it is essential to select a cost-effective and expeditious methodology while still providing satisfactory accuracy. We decided to utilize BEMT to analyze and choose the suitable propeller for the coaxial part of the subsystem. A detailed explanation of the development process of the BEMT for coaxial (and single) propellers and the validation of this method can be found in^18,29^.

**Figure 8:**
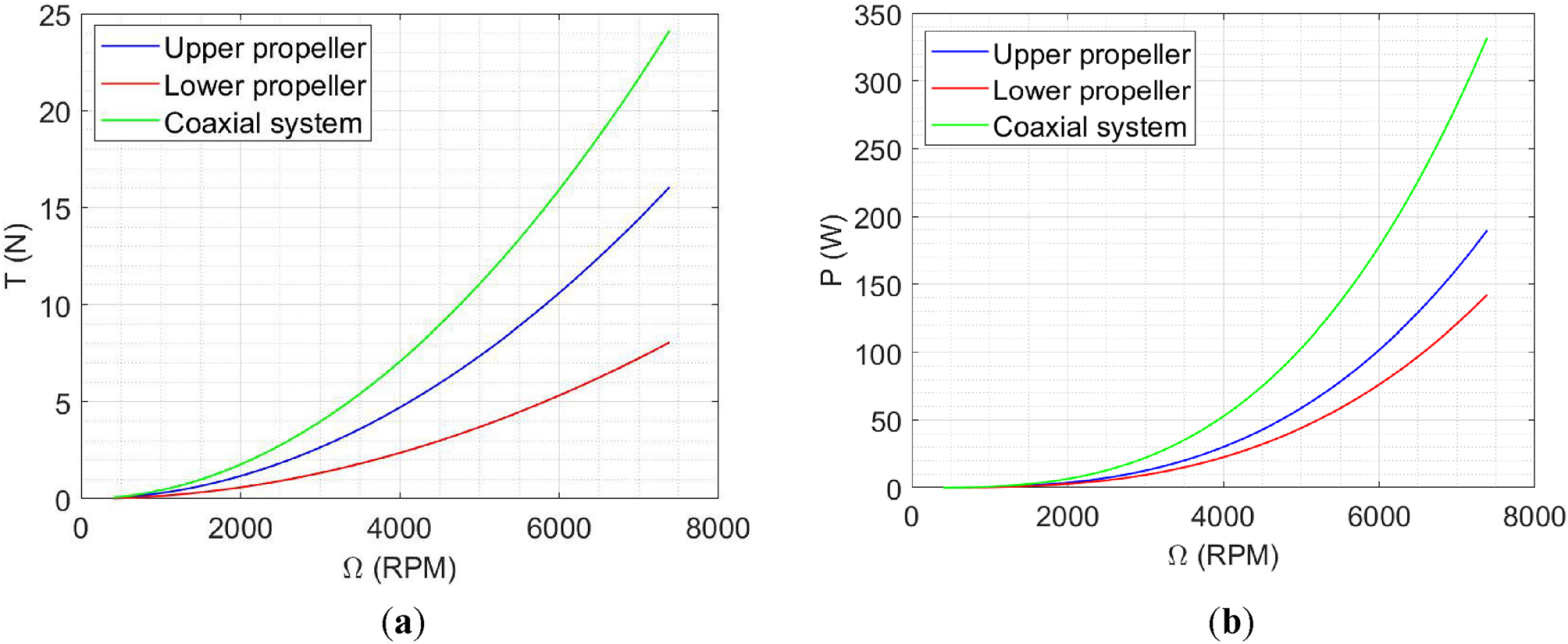
BEMT result for iFlight 12 × 4.5 propellers in Coaxial configuration: (**a**) Thrust; (**b**) power.

By employing the BEMT method from^18,29^, we can effectively simulate and evaluate various options for coaxial propellers. The selection of the most suitable choice is based on the requirements specified in section “System architecture design” and additional factors such as market availability, cost, and other relevant criteria. After evaluating different options, a pair of iFlight 12 × 4.5 propellers^56^ were selected. The result of BEMT analysis for the coaxial system (considering equal RPM for both upper and lower rotor) is illustrated in Fig. 8.

A comparative analysis of test data from various manufacturers was conducted to identify an appropriate control propeller. Based on the results of this investigation, the HQ3020 propellers were selected^51^.

Once the propellers have been selected, the next step is to identify brushless motors compatible with the chosen propellers. The requirements for the brushless motor include compatibility with the propeller and considerations such as efficiency, availability, cost, weight, and size, among others. After evaluating various options, Tmotor brushless motors were the most appropriate choice. Specifically, for the coaxial propellers, the Tmotor MN3510 KV700 was selected, while the Tmotor F1204 KV5000 was chosen for the control motor.

Following the selection of propellers and brushless motors, the next step is to choose appropriate ESCs. The primary considerations when selecting an ESC are voltage compatibility with the BLDC motor and sufficient capacity to handle the motor's maximum current. For the latter case, the maximum current of ESCs was selected to be at least 20% greater than the highest expected current draw of the relevant BLDC motor. The maximum current for Tmotor MN3510 KV700 and F1204 KV5000 motors are 19.4 A and 7 A respectively and both of them operate with 3S LiPo batteries with a maximum voltage of 12.6 V^51^. Therefore, the ESCs should be compatible with this voltage. Finally, the hobbypower 30 A^57^ and^58^ ESCs were selected for the main and control motors, respectively.

### Flight control subsystem design

As mentioned in the *system architecture design* phase, the flight control subsystem will be designed based on the PX4 autopilot, and the new configuration will be imported into the PX4 firmware. Therefore, in this stage, the hardware characteristics of this subsystem will be designed, and software development will be covered in *the hardware and software component design* phase (section “Component hardware and software design and manufacturing of the multirotor”). Because the developed autopilot firmware will be based on PX4 autopilot, it will be compatible with flight control boards and peripherals produced for PX4 firmware by different manufacturers. The Pixhawk1 autopilot and its necessary peripherals, such as GPS and telemetry module, were selected due to its low cost and availability in the local market^38^.

### Power supply subsystem design

The selected motors and ESCs in section “contro forces and moments generation subsystem design” require a voltage between 11.1 and 12.6 V. Therefore, a three-cell battery would suffice for this application.

To determine the required energy capacity, we need to consider each motor's required current and the multirotor hover endurance. To calculate the required current for control motors, using Tmotor established test result data for the F1204 KV5000 motor with HQ3020 propeller^51^, the current was calculated as 1.5 A. Still, for coaxial propellers, we need to use BEMT results. As mentioned earlier, BEMT can be used to calculate the required power for upper and lower propellers. These powers can then be used to calculate BLDC motors' consumed power and drawn current considering motors' efficiency. In hover flight, to have a trimmed flight, upper and lower propellers should generate a sufficient amount of thrust, and the sum of their yaw moment should be zero, i.e.:

4 $$T_{u} + T_{l} = 9.72 N .$$

5 $$M_{u} + M_{l} = 0 \;{\text{N}}\;{\text{m}} .{ }$$

These requirements should be met around 50% to 60% of throttle. The BEMT code can be employed to solve the system of equations graphically or numerically. The resulting speed for upper and lower propellers in hover flight is:

6 $$\omega_{u} = 4400{\text{ RPM}} .$$

7 $$\omega_{l} = 4870{\text{ RPM}} .$$

At this speed, the consumed power of the upper and lower rotors is 40.2W and 40.8W, respectively. The consumed power of MN3510 KV700 with these propellers can be calculated with the following equation:

8 $$P_{BLDC} = \frac{{P_{Propeller} }}{{\eta_{M} }} .$$

And $\eta_{M}$ is motor efficiency coefficient. For MN3510 KV700 after investigating published test result data from^39^, the value of $\eta_{M}$ was calculated as 0.85. Using the above equation, the required power for upper and lower motors can be calculated as 47.3 W and 48 W respectively. The electrical power of a BLDC motor can be calculated as follows:

9 $$P_{BLDC} = I_{A} \times V .$$

In which $I_{A}$ is the current and $V$ is the voltage. BLDC motors will experience a range of available voltage during the flight due to the discharging of the LiPo battery. The average voltage of 11.85 V could be considered for 3S batteries based. Considering the powers calculated above for the upper and lower motor and the voltage, each motor's drawn current can be calculated. The drawn currents for the upper and lower BLDC motors are 3.99 A and 4.05 A, respectively.

Finally, the total drawn current from the LiPo battery in hover flight is:

10 $$I_{{A_{{total~Hover}} }} = 4~ \times ~1.5 + 3.99 + 4.05 = 14.04~A~.$$

The required hover endurance is 15 min; therefore, the Ah can be calculated as:

11 $$Ah_{Hover} = 14.04 \times 0.25 = 3.51\;{\text{Ah }}.$$

Based on requirements in section “Requirement analysis”, the LiPo battery should not be discharged below 25–20% of its maximum capacity. Considering this criterion, the required capacity for LiPo battery is:

12 $$Ah_{LiPo} = 3.51 \times 1.25 \approx 4300 {\text{mAh }}{.}$$

The next requirement is maximum discharge rate; this parameter is specified with a C-rate, which, when multiplied by their capacity, indicates the safe maximum current that can be drawn from the LiPo battery without causing damage to it. The maximum current of MN3510 KV700 and F1204 KV5000 BLDC motors are 19.8 A and 7 A, respectively. Therefore, the maximum current drawn from the LiPo battery is 67.6 A. Considering the calculated Ah for the battery, the C-rate can be calculated as follows:

13 $$CR = \frac{67.6}{{4.3}} \approx 16\frac{1}{h}.$$

Therefore, a 3S LiPo battery with 4300 mAh capacity and (at least) 16 1/h C-rate is required. After investigating different available products, the Gens-Ace 3S 4300 mAh with 60C is selected.

For other components of this subsystem, i.e., the power distribution board and power module, the wiring of the multirotor subsystem should be considered. The most critical subsystem in this investigation is the flight control subsystem, specifically the autopilot board. Because it determines how different electric parts should be connected. Considering the Pixhawk1 board, the 8-channel CRIUS power distribution board and APM power module were selected^38^.

### Airframe subsystem design

This subsystem's requirements and overall shape are determined in *the system architecture design* stage. In this step, the more detailed design of this subsystem is carried out. The final design for each component of this subsystem which will lead into manufacturing plans and drawings will be carried out in *the hardware and software component design phase* (section “Component hardware and software design and manufacturing of the multirotor”).

**Figure 9:**
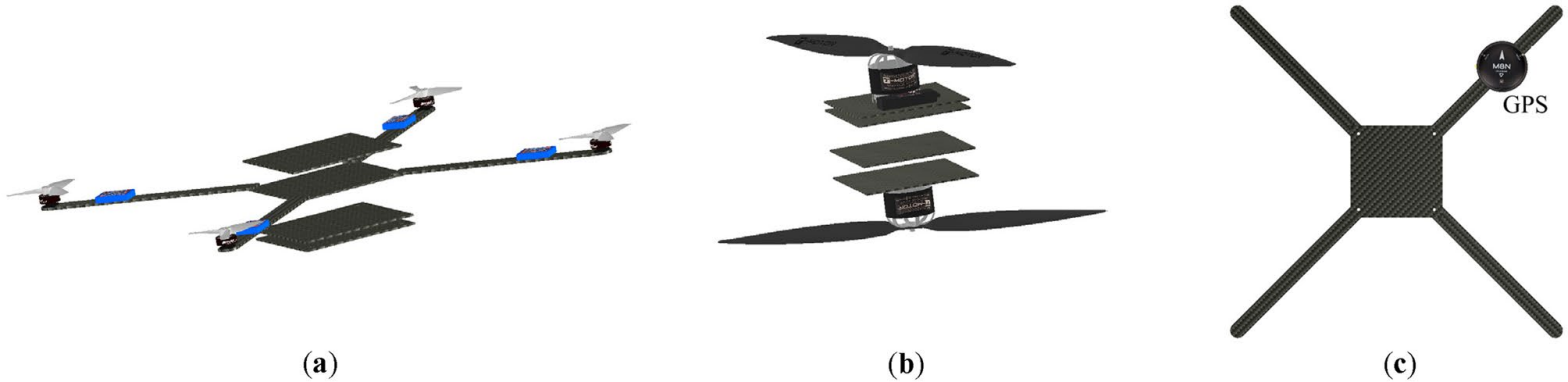
Examples of placements of different components: (**a**) control motors, propellers, and ESCs; (**b**) coaxial motors, propellers, and ESCs; (**c**) GPS module.

**Figure 10:**
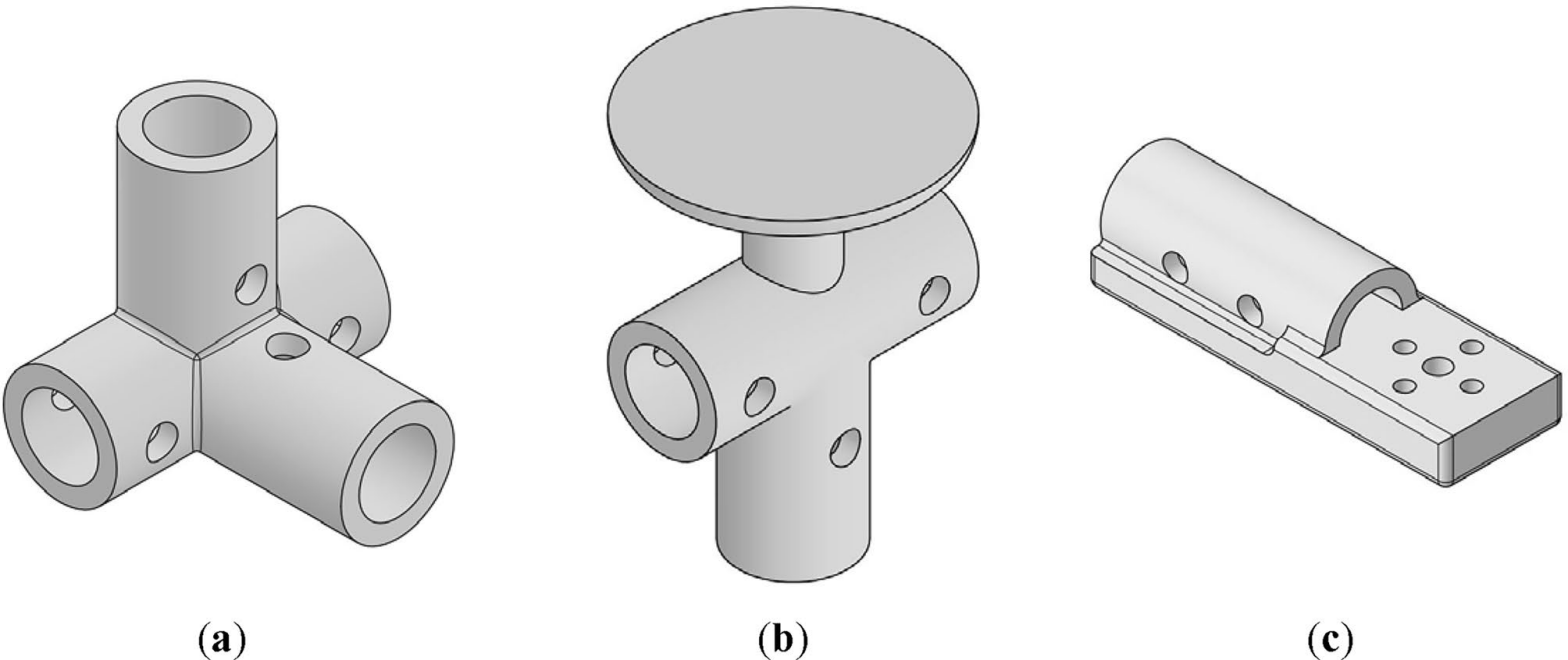
(**a**) Four-way connector to connect landing skids to payload holder bars; (**b**) connector to connect arm to landing skid with placement for GPS module; (**c**) control motor mount.

**Figure 11:**
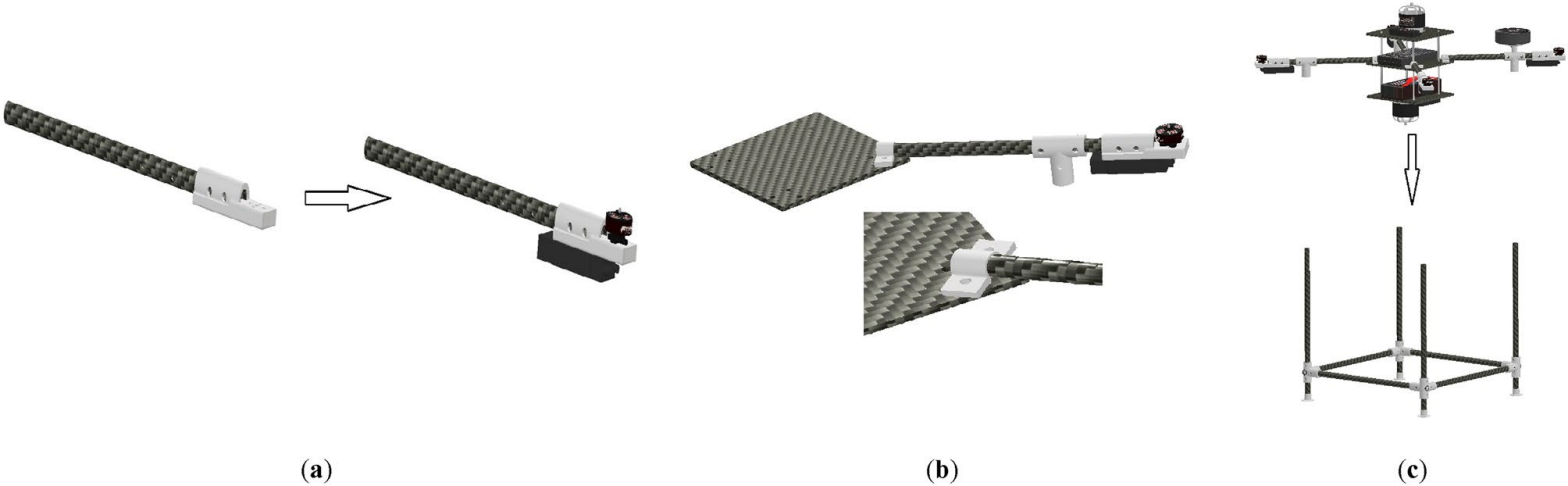
Examples of assembly process: (**a**) control motors and ESCs to arm; (**b**) arms to main plates; (**c**) landing skids and payload bars to main plates and arms.

The design process includes three main steps:

- The first step is to finalize the placement of different subsystem and their components. In Fig. 9 different example of placement is illustrated.

- After finalizing these decisions, the necessary and overall design parameters and decisions should be considered. For Example: the design of different connectors and how different components should be connected to each other should be made at this step. For example, the arm to landing skid connector or arm to control BLDC motors. Some examples are illustrated in Fig. 10.

- The final step is to investigate the assembly process of the multirotor and make some modifications to the design to make the assembly process as simple as possible and consider important factors such as ease of access to the LiPo battery to power on and power off the multirotor, etc. Some examples are illustrated in Fig. 11.

**Figure 12:**
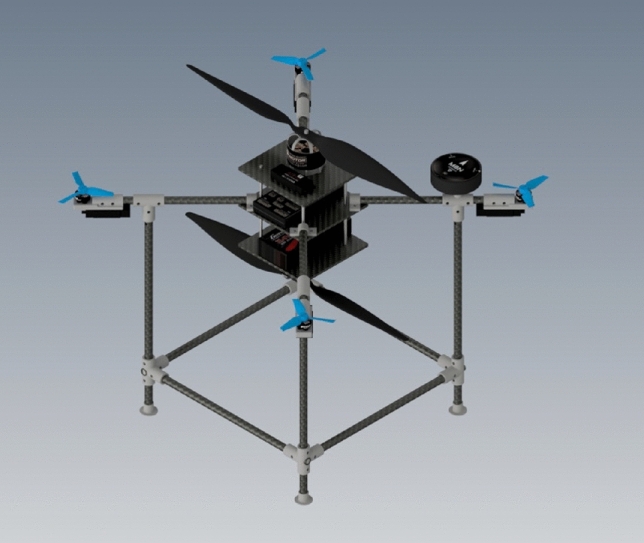
Final CAD model of the multirotor.

The airframe contains three plates between propellers that different parts like autopilot board will be installed on. When the slipstream of the upper propeller encounters these plates and components installed on them, the flow will get disturbed, and the overall efficiency will be reduced. To prevent this effect, a cover was added to the design with a better aerodynamic shape, improving the aerodynamics of coaxial propellers. The CAD model of the multirotor is illustrated in Fig. 12.

### Evaluating design cycle convergence

Finally, by utilizing the Eq. (3) and comparing them with the estimated design weight from the beginning of the design cycle, the convergence of the design cycle may be examined. It is worth mentioning that an additional weight of 50 g was considered for parts like bolts, wires, connectors, etc. The summation of the weight of different parts of subsystems is mentioned in Table 7.

**Table 7:** The weight summation of the multirotor components.

Component	Number	Total weight (g)
Coaxial motor	2	236
Control motor	4	28
Coaxial propeller	2	29
Control propeller	4	4
ESC for coaxial motor	2	53
ESC for control motor	4	32
Li-Po battery	1	250
Power distribution board	1	8
Power module	1	28
Autopilot Board	1	38
GPS module	1	32
Telemetry module	1	19
Airframe	1	200
Peripherals	-	50
	Sum (g)	1007

Using Eq. (3), the $W_{T.O}$ of the multirotor could be calculated as:

$$W_{T.O} = 1007 + 200 = 1207g$$

The estimated weight in section “System architecture design” was 1200 g; therefore, the design loop converged with less than 1% error. (Only the last cycle is mentioned here; the design cycle convergence is rapid, and in the current design, the convergence was obtained after three cycles).

## Component hardware and software design and manufacturing of the multirotor

In this stage, based on the development process determined for each subsystem in *the system architecture design phase*, *the flight control subsystem* and *the airframe subsystem* components will be designed in detail. In the current study, there is no need for a more detailed design for other subsystems' components.

### Software design of flight control subsystem

As mentioned before, developing the flight control subsystem includes importing the novel configuration of the multirotor into the PX4 firmware. Three files must be created to define a new configuration to the PX4 firmware: a geometry file, a mixer file, and an airframe file.

- Geometry file: The main geometric specifications of the multirotor, along with the thrust and torque coefficients, are determined in the geometry file. Some examples are propellers' position according to the body-fixed coordinate system, axis, and direction of rotation for each propeller, thrust and yaw moment coefficients.

Every geometry file has its specific key name, which will be used in the mixer file. Examples of geometry files can be found in^59^.

- Mixer file: The mixer file sets parameters to translate the controller output to each actuator command that controls motors (or servos if applicable). Different types of mixers are defined in PX4 firmware^59^; here, we used a “multirotor mixer” file. In a multirotor mixer file, the most important part is declaring the relevant geometry by its key and scaling roll, pitch, and yaw parameters compared to thrust output. Some examples of mixer files can be found in^59^.
- Airframe file: The airframe file contains the relevant parameters for the airframe, such as control coefficients, PWM signal ranges, number of battery cells used, sensor performance parameters, desired mixer, and settings for the order of the mixer outputs. The airframe file's most important aspect is configuring the mixer and specifying the order of the relationship between each mixer output and the corresponding PWM output for the autopilot. Adjusting other parameters is secondary and can be postponed until flight tests are conducted. If necessary, these parameters can be modified in the ground station software. Some examples of airframe files can be found in^59^.

### Hardware design of the airframe subsystem

This phase includes:

- Creating manufacturing plans and accurate drawings for different airframe parts, including plates, arms, landing skids, connectors, etc. In this step, a detailed drawing of each component should be prepared with exact dimensions, tolerances, thicknesses, etc. The blueprint might be necessary for carbon fiber composite, but for connectors, due to the 3D-print process, the STL CAD files should be generated.

**Figure 13:**
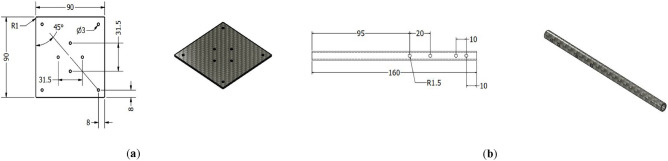
Examples of Manufacturing drawings for airframe components: (**a**) main plate where main motors will be installed; (**b**) arm with holes to place connectors for landing skid and control motor.

Figure 13 illustrates the drawing for main plates with placement for main motors and arms.

- Determining the manufacturing process for each component and determining necessary materials, tools, etc. For example, the CNC (or manual) milling machine for carbon plates might be used for cutting and creating holes, but for tubes, the cutting can be done with iron saws.

### Manufacturing the multirotor

**Figure 14:**
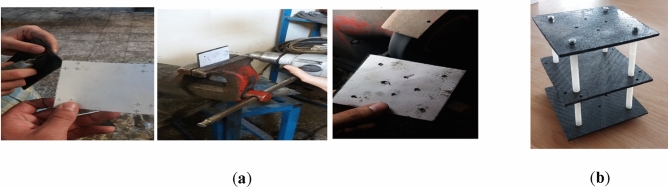
Manufacturing process for main plates: (**a**) cutting and creating holes; (**b**) main plates connected to each other using spacers.

**Figure 15:**
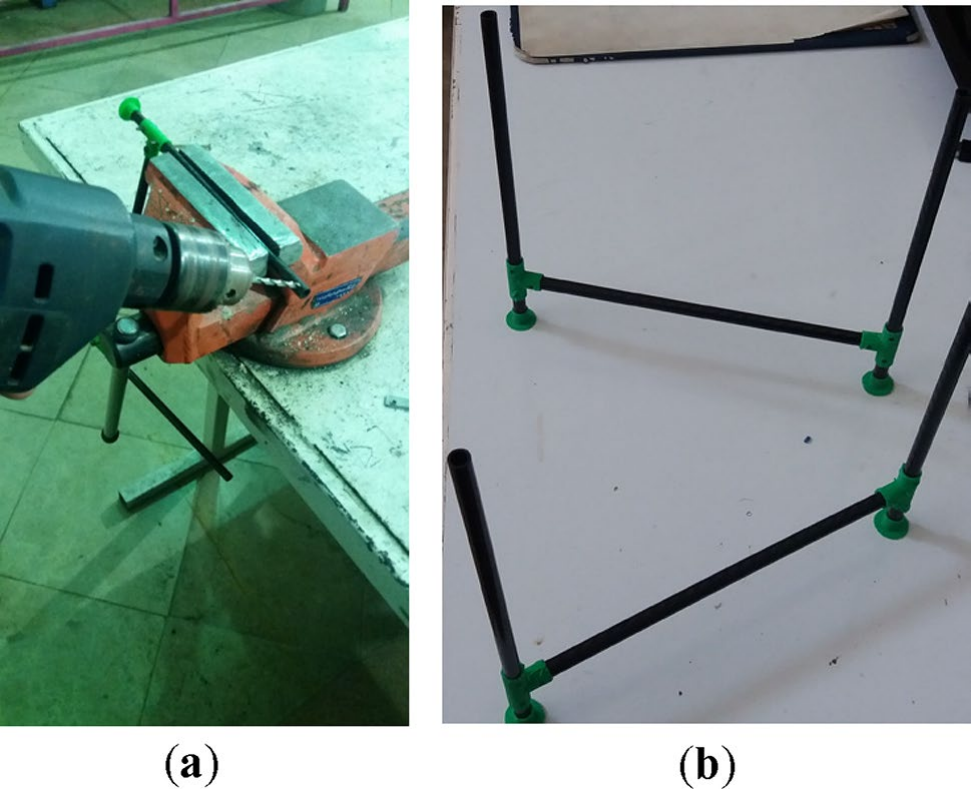
Manufacturing process for arms, landing skids and payload bars: (**a**) cutting and creating holes; (**b**) landing skids and payload bars are connected to each other.

**Figure 16:**
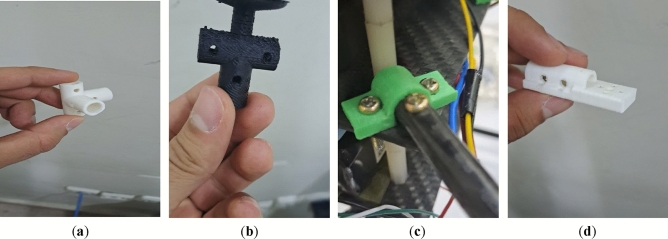
Some examples of 3D-printed parts: (**a**) four-way connectors to connect landing skids to payload bars; (**b**) connector to connect arm to landing skid with placement for GPS module; (**c**) arm to main plate connector; (**d**) control motor mount.

The first step of manufacturing the multirotor is preparing and developing different components and subsystems and making them ready to assemble. The most crucial subsystem in this step is the airframe subsystem, which needs a complete manufacturing process. For example, cutting and shaping carbon-epoxy plates using manual milling and creating necessary holes (Fig. 14), cutting carbon-epoxy composite tubes using a saw for arms, landing skids and payload holder bars and creating necessary holes (Fig. 15), 3D-printing of different connectors (Fig. 16), etc.

**Figure 17:**
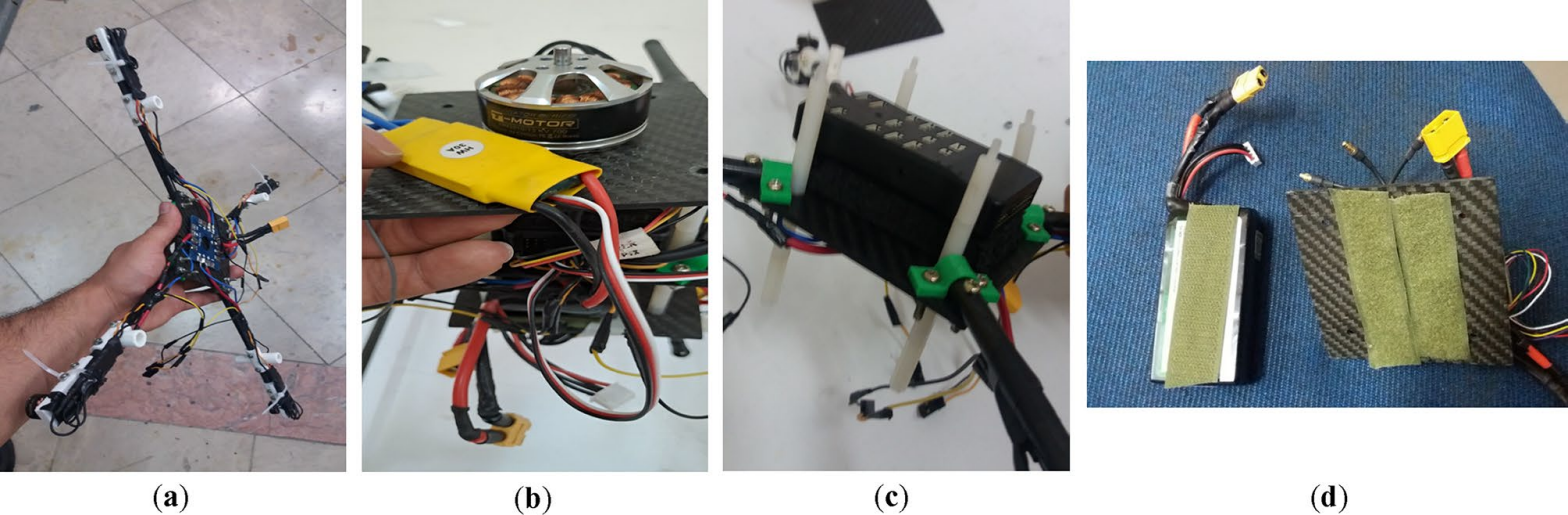
Some examples of assembly process: (**a**) assembly of arms, main plate, control motors and ESCs, and power distribution board; (**b**) assembly of main motor, Esc and main plate; (**c**) mounting Pixhawk1 to main plate; (**d**) using Velcro to connect LiPo battery to the main plate.

**Figure 18:**
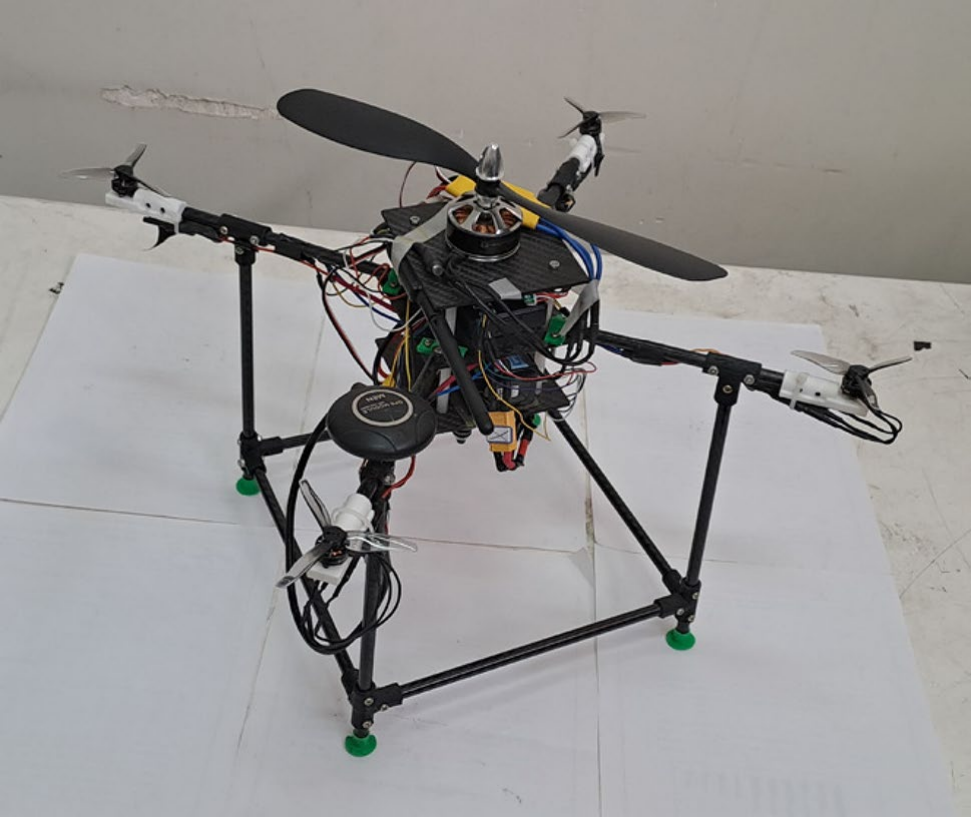
The manufactured multirotor.

After manufacturing of the airframe subsystem, the next step is integrating all other subsystems into the airframe and making the multirotor ready for flight. The assembly process is determined in the subsystem design phase (section “Subsystem design”). Some example of assembly steps is illustrated in Fig. 17. The multirotor is illustrated in Fig. 18.

## Component analysis and validation

As mentioned in section “Overall Design and development procedure”, in this stage, different components from different subsystems should be analyzed. In the case of this study, the most essential component is the PX4 firmware developed as the primary software component of the flight control subsystem. In this section, the analysis of this component is presented.

After the modifications on the PX4 firmware were carried out, we need to evaluate the modified firmware. Based on^38^, two sets of tests are performed to examine the performance of the flight control subsystem: software in the loop (SITL) and hardware in the loop (HILT). In SITL, the multirotor simulation is performed on a host computer. In this scenario, all multirotor subsystems are simulated on the computer, and the autopilot code is also executed on the host computer. In HITL, various multirotor subsystems are removed from the virtual environment and placed next to the host computer as hardware components. Typically, the first subsystem implemented as a hardware component is the autopilot board, and the first component examined is the autopilot board and its implemented firmware.

In SITL and HITL, the performance of the multirotor is evaluated, and adjustments of control coefficients, parameters, etc., can also be made in the simulation environment. Since assessing the autopilot's performance is crucial, only this component is implemented as hardware in the HITL simulation.

**Figure 19:**
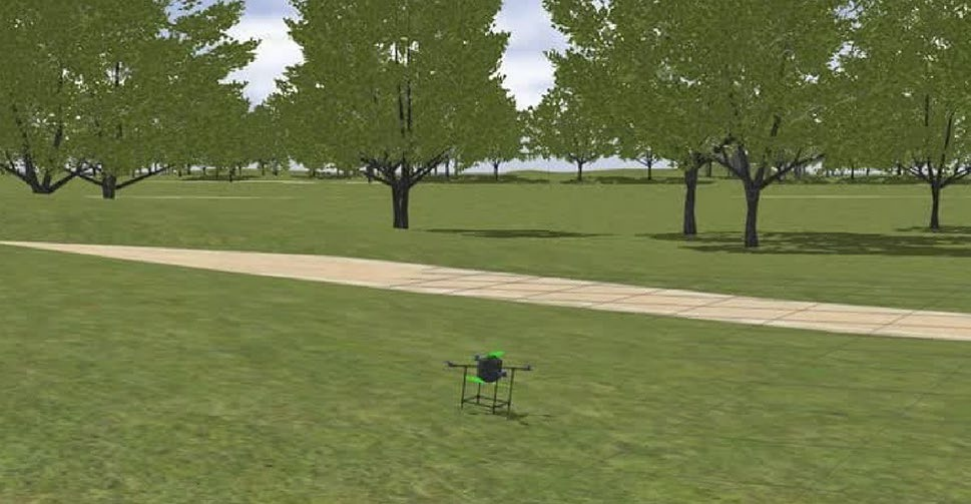
The multirotor model developed in GAZEBO.

There are different options for developing the model of the multirotor for SITL and HITL simulation purposes; the GAZEBO simulator^60^ is recommended by PX4 developers^23^ due to its ease of model development, ease of conducting different simulations, especially the HITL, as well as the open-source nature of this software, are among the reasons why it is recommended. The multirotor model was developed in the GAZEBO environment based on examples in^59^ and GAZEBO documents in^60^; the model is illustrated in Fig. 19.

### SITL simulation

**Figure 20:**
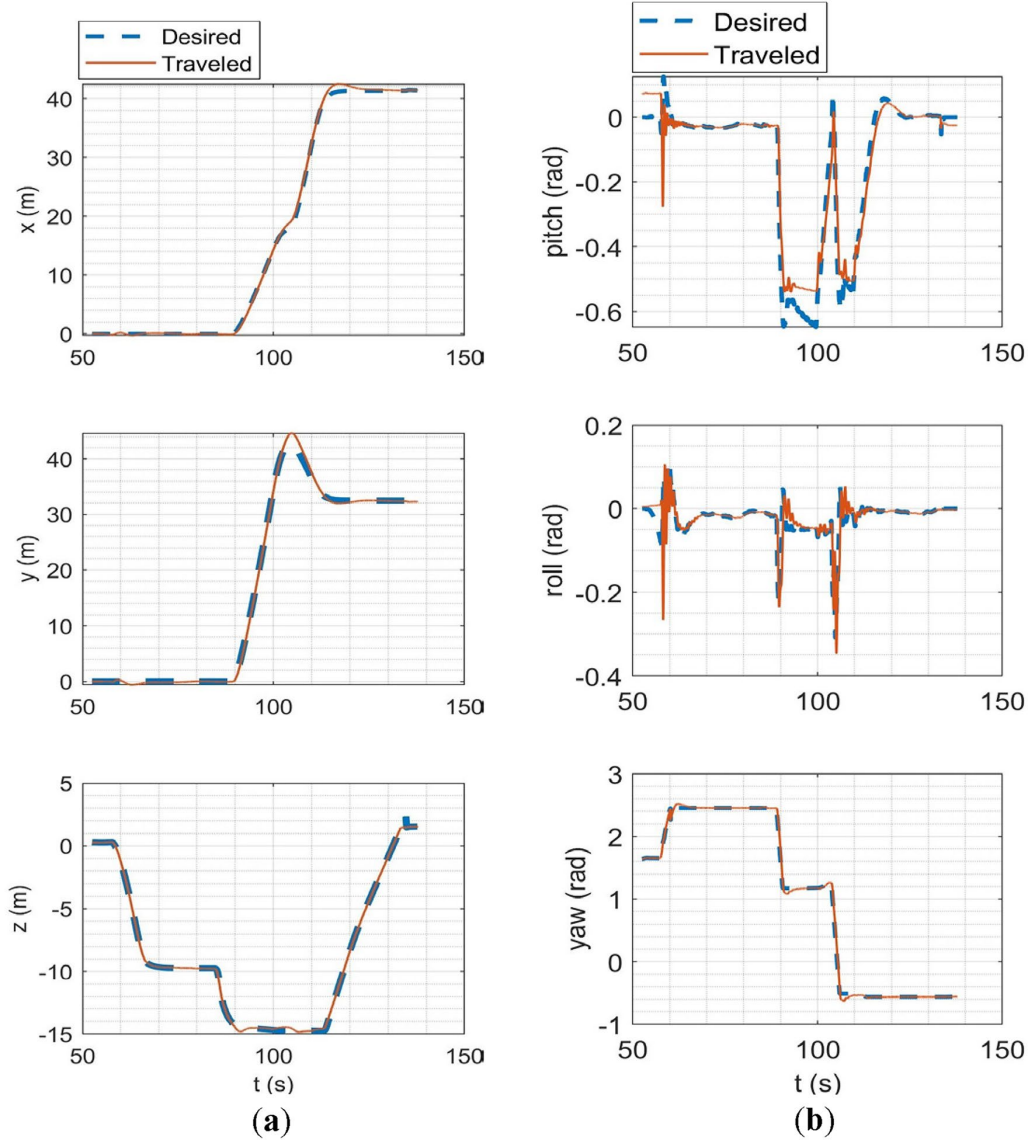
SITL simulation results: (**a**) tracking of desired trajectories in each axis of the inertial coordinate system; (**b**) tracking of Euler angles.

In the SITL simulation, the mission flight mode was simulated. In this scenario, the multirotor takes off from the ground to a desired altitude (30 m) and then starts following a path between the user's desired waypoints. The ground control station software used in this simulation was Qgroundcontrol^61^. The tracking of desired trajectories in each axis of the inertial coordinate system and the tracking of desired Euler angles are shown in Fig. 20.

### HITL simulation

**Figure 21:**
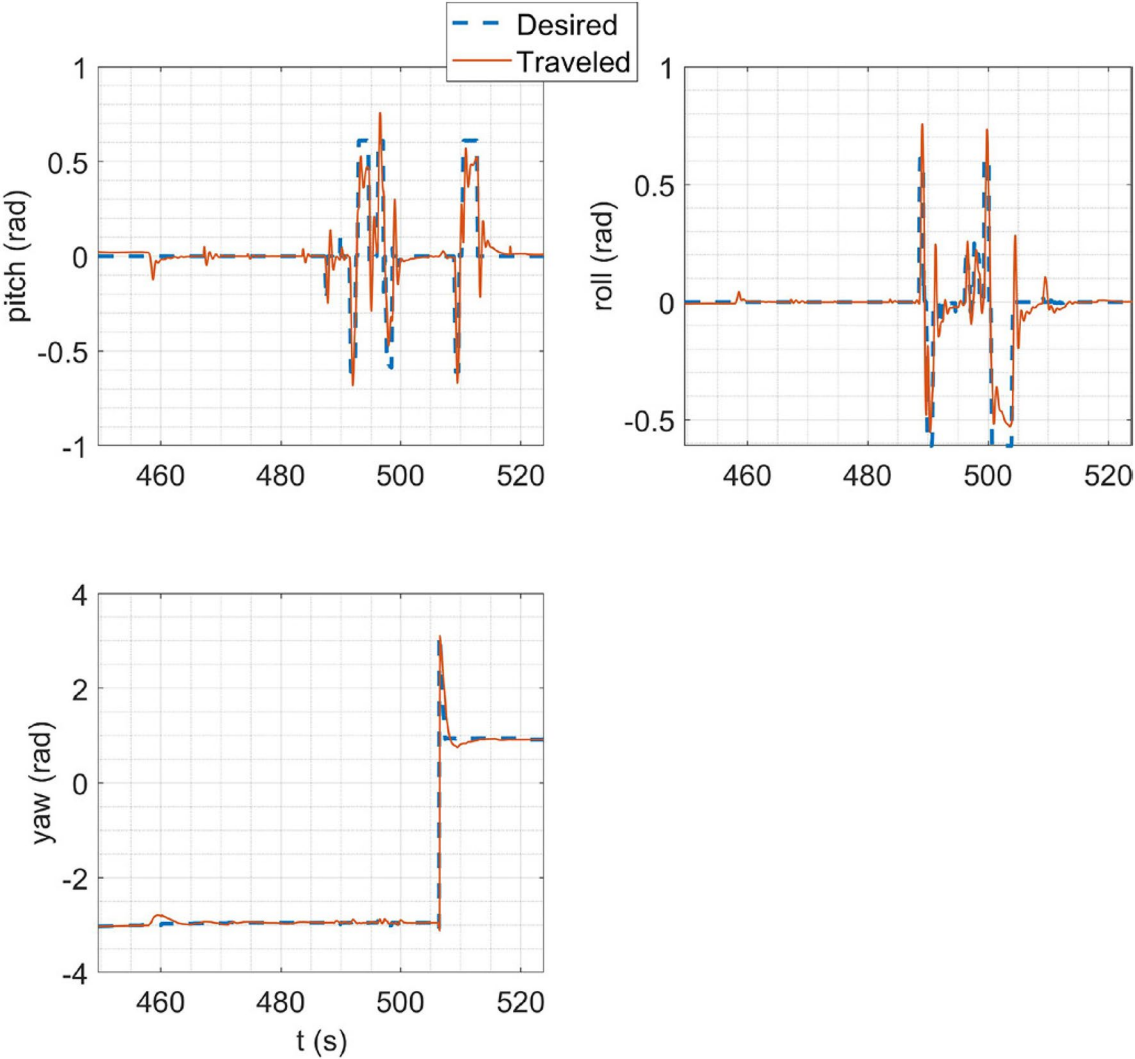
Tracking of desired Euler angles in stabilize flight mode in HITL.

**Figure 22:**
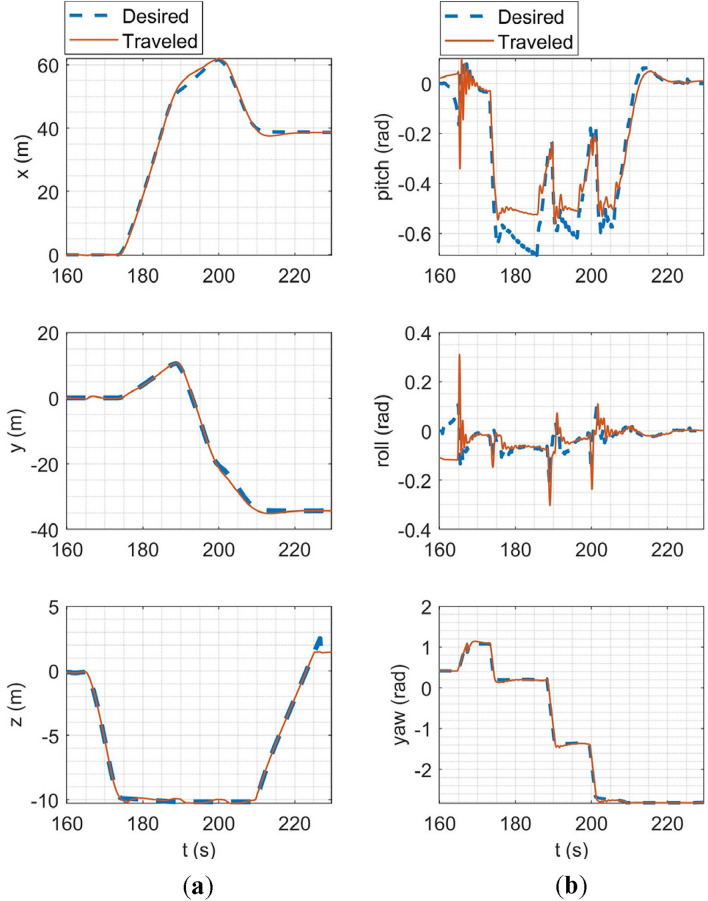
HITL mission mode simulation results: (**a**) tracking of desired trajectories in each axis of the inertial coordinate system; (**b**) tracking of Euler angles.

In HITL simulation, the stabilize flight mode was first simulated in which the multirotor tries to follow the desired roll and pitch angles alongside the desired yaw rate and vertical speed commands from the RC transmitter. Also, the vehicle tries to maintain its attitude if the sticks are released. Tracking of desired Euler angles is illustrated in Fig. 21. Next, the mission mode was simulated with the same procedure carried out in the SITL simulation but with the difference in desired waypoints. Tracking of desired trajectories in each axis of the inertial coordinate system and tracking of desired Euler angles are illustrated in Fig. 22.

The overall performance of the multirotor is acceptable. In linear position tracking in auto mode, the maximum steady state error is about 1% with the maximum settling time of 2 s and 1% overshoot. In attitude channels, the maximum steady state error is about 1% with the settling time of 1.1 s and a maximum of 5% overshoot.

## Subsystem analysis and validation

As mentioned in section “Overall design and development procedure”, this stage of development includes a more comprehensive analysis of the multirotor from different aspects and improving the design of subsystems. In the current study, three main aspects of the multirotor are analyzed: aerodynamic, flight dynamic, and structure.

### Aerodynamic analysis

Two important aerodynamic phenomena require further analysis: the coaxial propeller's thrust force and the multirotor drag.

#### Coaxial propellers analysis

While the BEMT method employed in the subsystem design provides adequate accuracy and precision, it is recommended to utilize more advanced analysis methods, such as CFD, to enhance design reliability and comprehensively analyze propeller performance, including the effects of propeller distance and airframe plates' blockage. The CFD simulation of coaxial propellers involves creating a 3D CAD model of the coaxial propeller. A rotating disk around each propeller and a larger stationary area around the whole coaxial system were defined as simulation boundaries, with their dimensions selected based on^31^. As mentioned in section “Subsystem design”, a cylindrical body with conic ends was added to the design to cover the space between the upper and lower propellers.

**Figure 23:**
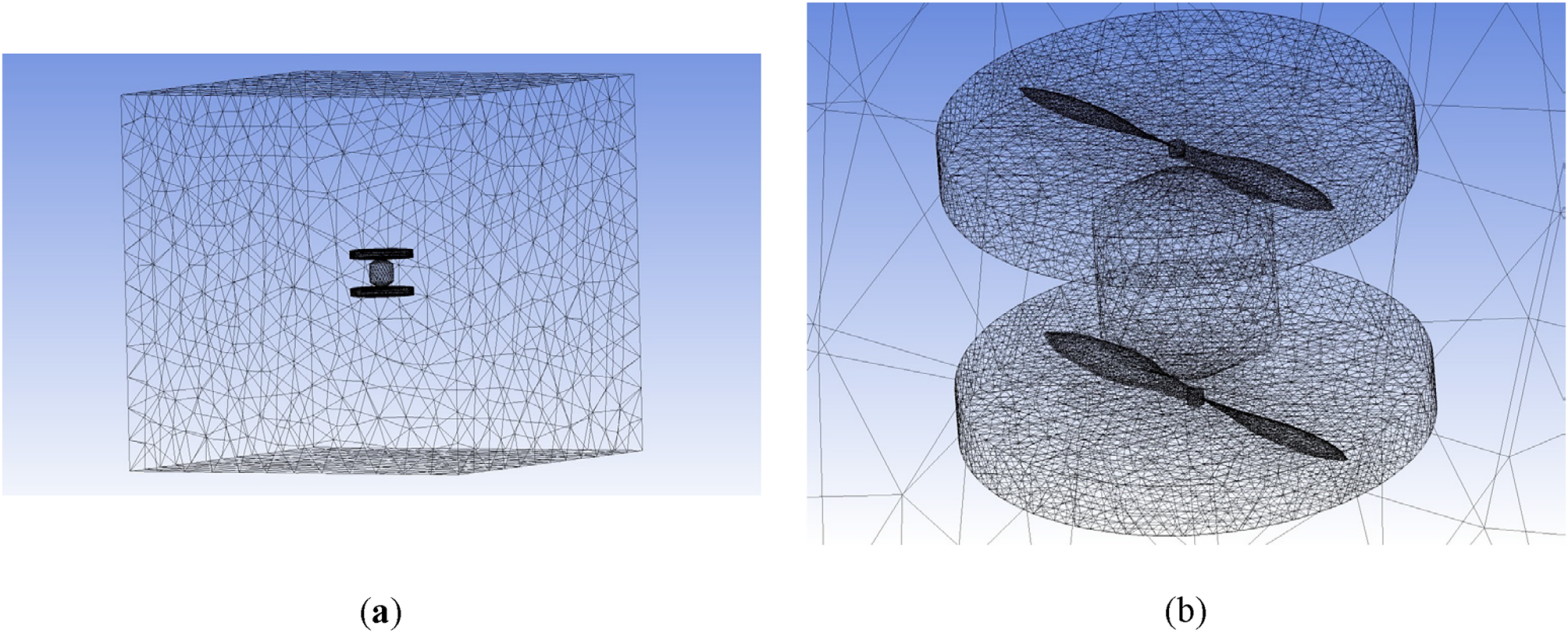
Coaxial propeller model and meshing: (**a**) the entire domain, (**b**) the coaxial system.

A tetrahedral mesh with 400,000 elements was generated. Various parameters were examined to evaluate the mesh's adequacy, including skewness and aspect ratio^36^. The average aspect ratio for this mesh was 1.8, with a standard deviation of 0.48, which is considered acceptable based on^20^. The average skewness was 0.24 with a standard deviation of 0.12, also within acceptable limits^20^. The coaxial system model is illustrated in Fig. 23.

**Figure 24:**
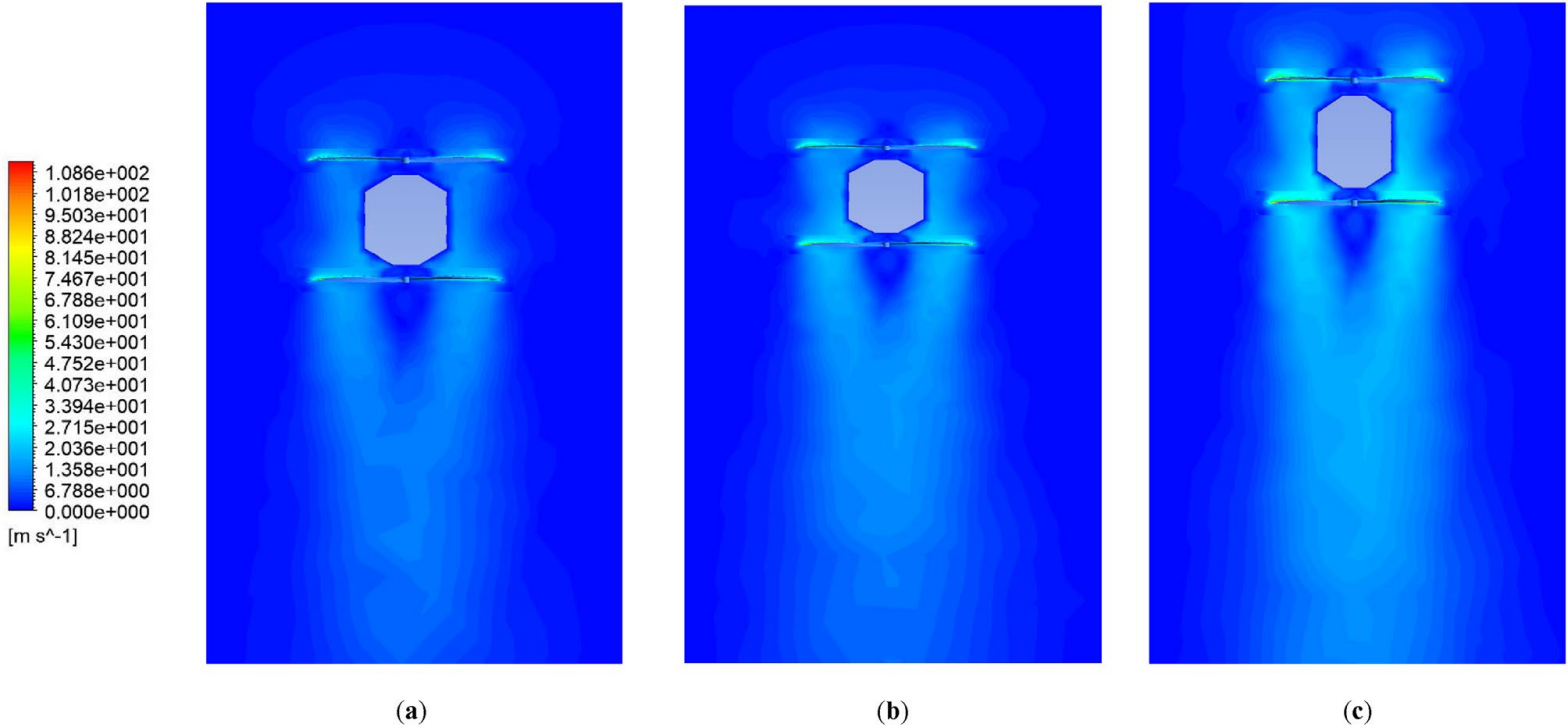
Velocity contour of coaxial propellers: (**a**) 4500 RPM; (**b**) 5550 RPM; (**c**) 7000 RPM.

**Figure 25:**
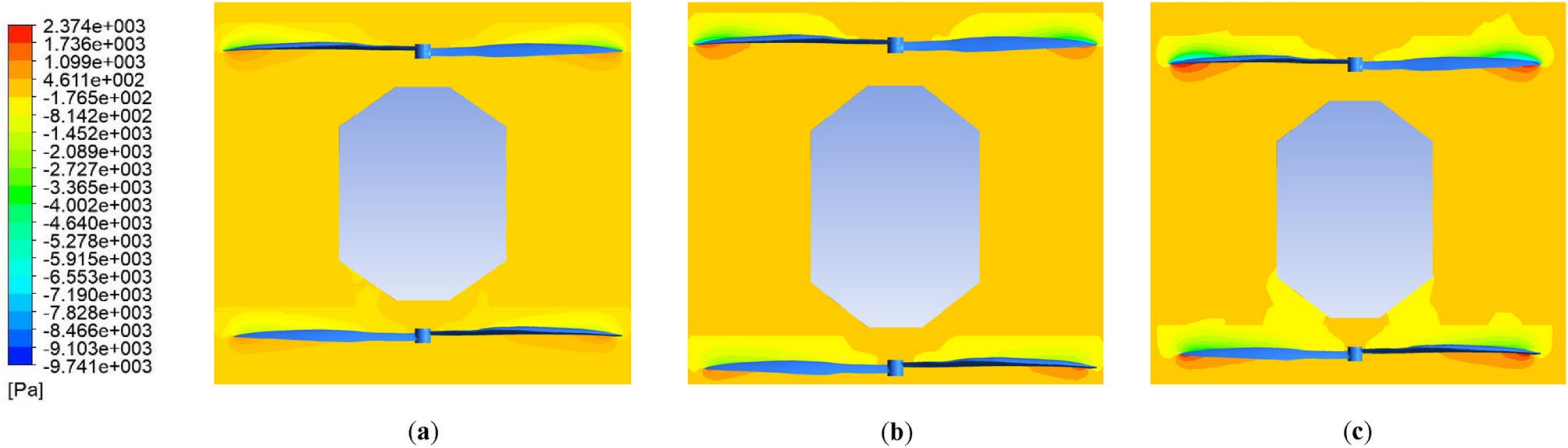
Pressure contour of coaxial propellers: (**a**) 4500 RPM; (**b**) 5550 RPM; (**c**) 7000 RPM.

The k-ω SST turbulence model was selected, which has been utilized in different research in analysis propellers, multirotor and coaxial propellers due to its accurate prediction of turbulence model, handling complex geometries, computational efficiency, etc. Also, each rotating area's rotational speed around the propellers was specified. The simulation was then conducted with a time step of 0.5 s for 100 time steps. A combination of different revolution speeds (equal speed for upper and lower propellers) from 4500 to 7000RPM with different axial velocities from − 3 to 3 m/s was conducted. The velocity and pressure contours for the coaxial system in three different RPMs are illustrated in Figs. 24 and 25, respectively.

**Figure 26:**
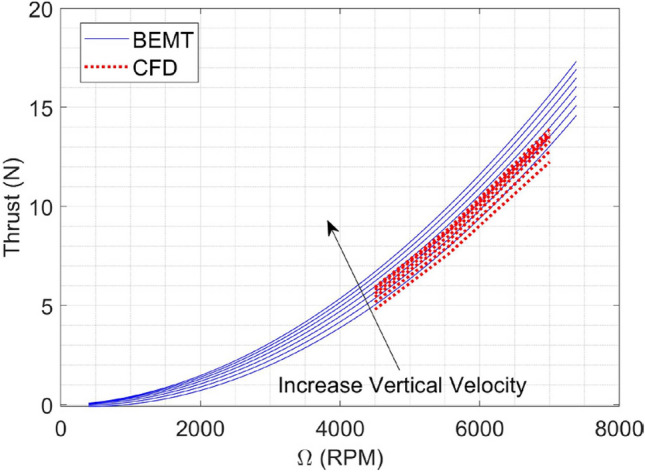
BEMT and CFD results for upper propeller Thrust force.

**Figure 27:**
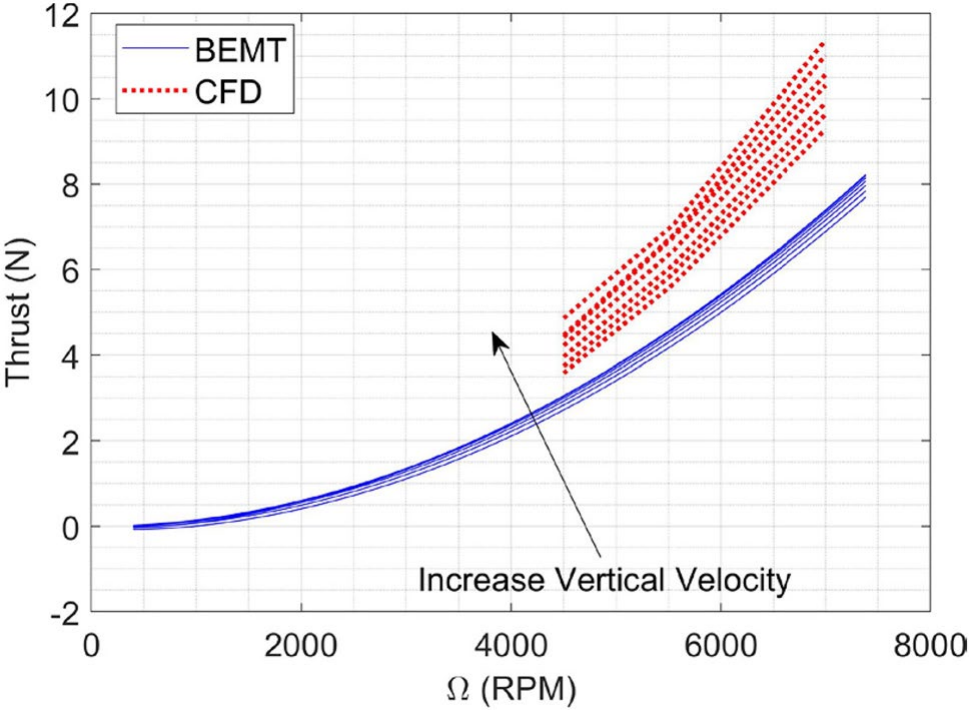
BEMT and CFD results for lower propeller: (**a**) Thrust; (**b**) Yaw moment.

A comparison between BEMT and CFD results for the upper and lower propeller is shown in Figs. 26 and 27, respectively. The results indicate that for the upper propeller, there is no significant difference between BEMT and CFD analysis in the thrust force prediction, and the BEMT method results utilized in *the subsystem design* is valid and also can be used in 6DOF modeling and simulation with no need for modification. However, for the lower propeller, there is a considerable difference between BEMT and CFD predictions. CFD analysis predicts an average of 30% more thrust force than BEMT methods estimated. The observed difference in the performance may be attributed to several factors, including the spacing and the cover between propellers.

An important result from both CFD and BEMT analyses is that with increasing axial speed over the propellers (climbing flight), the axial flow velocity over the inner section of the lower propeller increases. This increase in velocity leads to a reduction in effective angle of attack and a decrease in thrust produced by the lower propeller. This issue is one of the challenges of this configuration, limiting the attainment of higher climb speeds.

#### The multirotor drag

In^33^, a comprehensive study is carried out on the multirotor's drag force and drag-liked aerodynamic effects. This reference divides the multirotor drag force into five main subsets:

- Blade flapping drag: When moving through the air, propellers generate an unequal force between the advancing and retreating blade sections due to the difference in velocity. This causes cyclic motion similar to flapping, diverting the direction of thrust force from vertical and creating a counteracting drag.
- Induced drag: Semi-rigid or fully rigid blades (like one used in most of the small multirotors) do not flap freely to create aerodynamic balance, causing the advancing blade to generate more lift than the retreating blade, resulting in a net instantaneous induced drag that opposes the apparent wind direction and is proportional to its velocity.
- Translational drag: When a multirotor is in transitional flight, the airflow over the disk is not purely vertical, as the incoming air is deflected towards the blade plane due to the blade's influence. This change in the momentum of the airflow results in an additional force known as the transitional force acting on the propeller system.
- Profile drag: This is caused by the propeller blades' transverse velocity as they move through the air.
- Parasite drag: This is the drag incurred as a result of the non-lifting surfaces of the multirotor.

A comprehensive CFD analysis that includes aeroelastic effects and a full-body analysis of the multirotor should be carried out to determine all mentioned subsets of drag force. Another approach that is suggested in^25,34^ is to consider a simple model for the drag force for 6DOF modeling and design procedures. These drag forces can become more accurate using flight test data and investigating the accelerometer sensor data.

Among the drag subsets mentioned above, the parasite drag is not dependent on the aeroelastic and transient behavior of the multirotor propellers and can be analyzed simply using CFD analysis. Therefore, this subset of drag was studied in this stage, and the modifications to consider other subsets of drag were postponed until after the flight tests.

**Figure 28:**
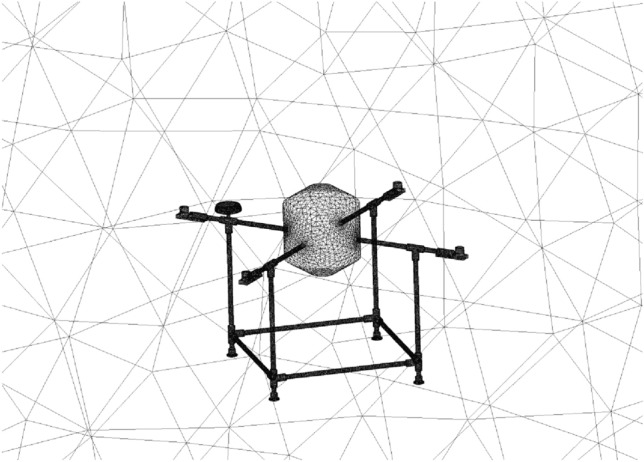
Multirotor body model and meshing.

CFD simulations were utilized for the calculation of body drag. Initially, the body geometry was modeled. Subsequently, the environment surrounding the body was enclosed in a 2 × 2 × 2 m cube. The domain was then meshed, creating 1,623,000 tetrahedral elements. As in the previous analysis, the aspect ratio and the skewness were employed to assess the mesh quality. The average aspect ratio of the mesh was 1.84 with a standard deviation of 0.46; additionally, the average skewness was 0.23 with a standard deviation of 0.12. The multirotor body model is illustrated in Fig. 28.

In contrast to the previous analysis, a steady-state solver was employed since the simulation had no blades. The k-ω SST turbulence model, proposed in reference^36^ for multirotor drag analysis, was utilized. However, other turbulence models like k-ε were also considered, and the simulation outputs did not differ significantly. The body-fixed coordinate system which is defined in section “Flight dynamic analysis” was considered to analyze the airframe drag in different directions. The flight simulation was conducted in three directions of the body-fixed coordinate system. In the (x) and (y) directions, 3, 7, and 10 m/s speeds were considered. In the (z) direction, 1, 2, and 3 m/s speeds were considered. Drag force is calculated as:

14 $$D = \frac{1}{2}\rho V^{2} SC_{D} .$$

In this equation, S is the reference area, and C_D_ is the drag coefficient. The reference area for the airframe was selected based on the cylinder part of the cover between the upper and lower propeller. The simulation was then executed for 1000 iterations. The solution converged appropriately, and the drag coefficients in different directions of the body-fixed coordinate system were calculated. The result is illustrated in Table 8.

**Table 8:** Drag coefficient of the airframe in body-fixed coordinate system.

Drag coefficient	Value
$C_{{Dx}}$	0.32
$C_{{Dy}}$	0.32
$C_{{Dz}}$	0.14

The payload is suspended under the coaxial system at a suitable distance in the designed airframe. The payload is considered a box with a 10 × 10 × 5 cm dimension, and the drag coefficient for the cube in sideway speed is equal to 1.07^62^.

### Flight dynamic analysis

The main assumptions for modeling the multirotor are listed below:

- The earth will be considered to be flat.
- The multirotor and its payload are assumed to be a single rigid body with no elastic behavior.
- The multirotor weight and moments of inertia will be constant during the flight.
- Forces that affect the multirotor are the thrust force of the propellers, drag force, and weight. Propellers control moment, gyroscopic moment, and the moment generated from the payload drag force are the moments affecting the multirotor. (The drag force of the multirotor is assumed to be applied to the center of gravity and therefore will have no moment)

The translational and attitude dynamic model equations can be derived based on^63^:

Translational dynamic:

15 $$\left[ {{\varvec{f}}_{T} ]^{B} + } \right[{\varvec{f}}_{A} ]^{B} + \left[ {{\varvec{f}}_{g} } \right]^{B} = m[ \left( {\frac{{d{\varvec{v}}_{B}^{I} }}{dt} + {\varvec{\omega}}^{{{\text{BI}}}} \times {\varvec{v}}_{B}^{I} } \right)]^{B} .$$

Attitude dynamic:

16 $$\left[ {{\varvec{M}}_{{\varvec{T}}} } \right]^{B} + \left[ {{\varvec{M}}_{{{\varvec{AP}}}} } \right]^{B} + \left[ {{\varvec{M}}_{{\varvec{G}}} } \right]^{B} = \left[ {{\varvec{I}}_{B}^{B} \dot{\user2{\omega }}^{{{\text{BI}}}} +{\varvec{\varOmega}}^{{{\text{BI}}}} {\varvec{I}}_{B}^{B} {\varvec{\omega}}^{{{\text{BI}}}} } \right]^{B} .$$

In these equations, ${\varvec{f}}_{T}$, ${\varvec{f}}_{A}$ and ${\varvec{f}}_{g}$ are thrust force vector, aerodynamic force vector and gravity force vector, respectively. ${\varvec{M}}_{{\varvec{T}}}$**,** ${\varvec{M}}_{{{\varvec{AP}}}}$ and ${\varvec{M}}_{{\varvec{G}}}$ are moment vectors from propellers thrust, aerodynamic drag of payload and gyroscopic effects, respectively. The translational and rotational velocity vectors are illustrated by ${\varvec{v}}$ and ${\varvec{\omega}}$, and ${\varvec{\varOmega}}$ is the skew-symmetric matrix form of ${\varvec{\omega}}$. The subscripts and superscripts $B$ and $I$ refer to body frame (or coordinate system) and Inertial frame (or coordinate system). ${\varvec{I}}_{B}^{B}$ is the matrix of moment of inertia of the multirotor in body frame and $m$ is the mass of the multirotor.

Equations (15) and (16) each provide three equations in each direction of a body-fixed coordinate system. This coordinate system is centered at the multirotor center of gravity, with the x-axis pointing forward, the y-axis pointing to the right, and the z-axis pointing downward.

The translational and attitude kinematic equations establish the relationship between the multirotor velocity in the inertial coordinate system and its velocity in the body-fixed coordinate system (u, v, w) and the relationship between the Euler angles ($$\phi , \theta , \psi$$) rate and the multirotor angular velocities ($$p, q,r)$$ in the body coordinate system, respectively.

17 $$\left[ {\begin{array}{*{20}c} {\dot{\varphi }} \\ {\dot{\theta }} \\ {\dot{\psi }} \\ \end{array} } \right] = \left[ {\begin{array}{*{20}c} {p + q\sin \phi \tan \theta + r\cos \phi \tan \theta } \\ {q\cos \phi - r\sin \phi } \\ {\left( {q\sin \phi + r\cos \phi } \right)\sec \theta } \\ \end{array} } \right]$$

18 $$\left[ {\begin{array}{*{20}c} {\dot{x}_{I} } \\ {\dot{y}_{I} } \\ {\dot{z}_{I} } \\ \end{array} } \right] = \left[ {\begin{array}{*{20}c} {{\text{cos}}\psi {\text{cos}}\theta } & {{\text{cos}}\psi {\text{sin}}\theta {\text{sin}}\phi - {\text{sin}}\psi {\text{cos}}\phi } & {{\text{cos}}\psi {\text{sin}}\theta {\text{cos}}\phi + {\text{sin}}\psi {\text{sin}}\phi } \\ {{\text{sin}}\psi {\text{cos}}\theta } & {{\text{sin}}\psi {\text{sin}}\theta {\text{sin}}\phi + {\text{cos}}\psi {\text{cos}}\phi } & {{\text{sin}}\psi {\text{sin}}\theta {\text{cos}}\phi - {\text{cos}}\psi {\text{sin}}\phi } \\ { - {\text{sin}}\theta } & {{\text{cos}}\theta {\text{sin}}\phi } & {\cos \theta {\text{cos}}\phi } \\ \end{array} } \right]\left[ {\begin{array}{*{20}c} u \\ v \\ w \\ \end{array} } \right]$$

The forces and moment in Eqs. (15) and (16) are expressed as follows^37^:

19 $$[{\varvec{f}}_{T} ]^{B} = \left[ {\begin{array}{*{20}c} 0 \\ 0 \\ { - \left( {T_{1} + T_{2} + T_{3} + T_{4} + T_{u} + T_{l} } \right)} \\ \end{array} } \right] .$$

20 $$\left[ {{\varvec{f}}_{A} } \right]^{B} = \left[ {\begin{array}{*{20}c} { - 0.5\rho \left| {u - u_{g} } \right|\left( {u - u_{g} } \right)s_{x} C_{{{\text{D}},{\text{x}}}} } \\ { - 0.5\rho \left| {v - v_{g} } \right|\left( {v - v_{g} } \right)s_{y} C_{{{\text{D}},{\text{y}}}} } \\ { - 0.5\rho \left| {w - w_{g} } \right|\left( {w - w_{g} } \right)s_{z} C_{{{\text{D}},{\text{z}}}} } \\ \end{array} } \right] + \left[ {\begin{array}{*{20}c} { - 0.5\rho \left| {u - u_{g} } \right|\left( {u - u_{g} } \right)s_{{x_{P} }} C_{{{\text{D}},{\text{x}}_{{\text{P}}} }} } \\ { - 0.5\rho \left| {v - v_{g} } \right|\left( {v - v_{g} } \right)s_{{y_{P} }} C_{{{\text{D}},{\text{y}}_{{\text{P}}} }} } \\ { - 0.5\rho \left| {w - w_{g} } \right|\left( {w - w_{g} } \right)s_{{z_{P} }} C_{{{\text{D}},{\text{z}}_{{\text{P}}} }} } \\ \end{array} } \right] .$$

21 $$\left[ {{\varvec{f}}_{g} } \right]^{B} = m\left[ {\begin{array}{*{20}c} { - g\sin \theta } \\ {g\sin \phi \cos \theta } \\ {g\cos \phi \cos \theta } \\ \end{array} } \right] .$$

22 $$[\user2{M}_{\user2{T}} ]^{B} = \left[ {\begin{array}{*{20}l} {L_{{arm}} \cos \left( {45^\circ } \right) \times ~\left( {T_{2} + T_{3} - T_{1} - T_{4} } \right)} \\ {L_{{arm}} \cos \left( {45^\circ } \right) \times ~\left( {T_{1} + T_{3} - T_{2} - T_{4} } \right)} \\ {M_{{z_{B} Tu}} - M_{{z_{B} Tl}} + M_{{z_{B} T1}} + M_{{z_{B} T2}} - M_{{z_{B} T3}} - M_{{z_{B} T4}} } \\ \end{array} } \right]~.$$

23 $$[\user2{M}_{{\user2{AP}}} ]^{B} = \user2{L}_{{\user2{armP}}} \times \user2{f}_{{\user2{AP}}} = \user2{~L}_{{\user2{armP}}} \times \user2{~~}\left[ {\begin{array}{*{20}c} { - 0.5\rho \left| {u - u_{g} } \right|\left( {u - u_{g} } \right)s_{{x_{P} }} C_{{{\text{D}},{\text{x}}_{{\text{P}}} }} } \\ { - 0.5\rho \left| {v - v_{g} } \right|\left( {v - v_{g} } \right)s_{{y_{P} }} C_{{{\text{D}},{\text{y}}_{{\text{P}}} }} } \\ { - 0.5\rho \left| {w - w_{g} } \right|\left( {w - w_{g} } \right)s_{{z_{P} }} C_{{{\text{D}},{\text{z}}_{{\text{P}}} }} } \\ \end{array} } \right]~.$$

24 $$[{\varvec{M}}_{{\varvec{G}}} ]^{B} = \left( {J_{CP} \left( {\omega_{3} + \omega_{4} - \omega_{1} - \omega_{2} } \right) + J_{MP} \left( {\omega_{l} - \omega_{u} } \right) } \right) \times \left[ {\begin{array}{*{20}c} q \\ { - p} \\ 0 \\ \end{array} } \right] .$$

**Figure 29:**
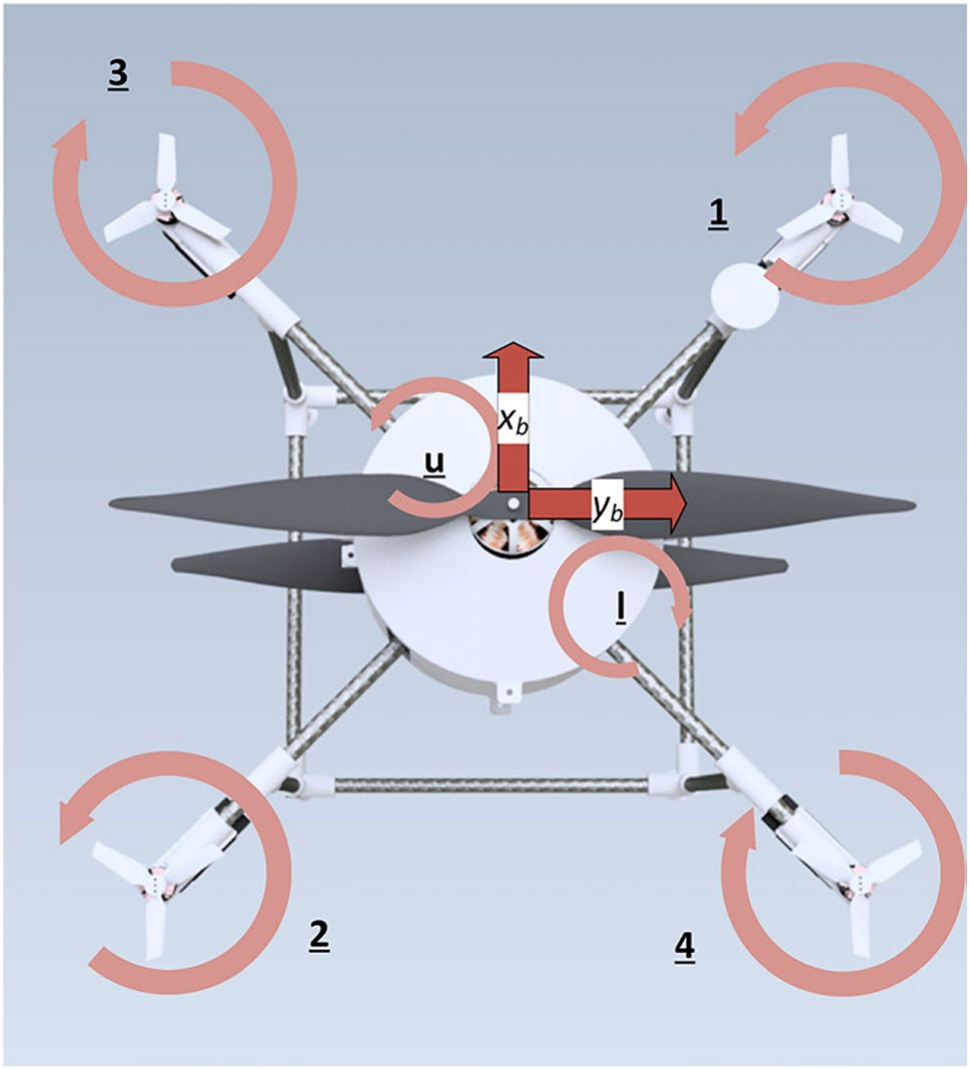
Propellers order and direction of rotation.

In the above equations, index 1–4 are propeller numbers based on Fig. 29, and “u” and “l” refer to the upper and lower propellers, respectively. For thrust and yaw moment, the BEMT is utilized considering the CFD analysis corrections. For the drag force, the drag analysis in section “The multirotor drag” can be used.

#### Controller design

**Figure 30:**
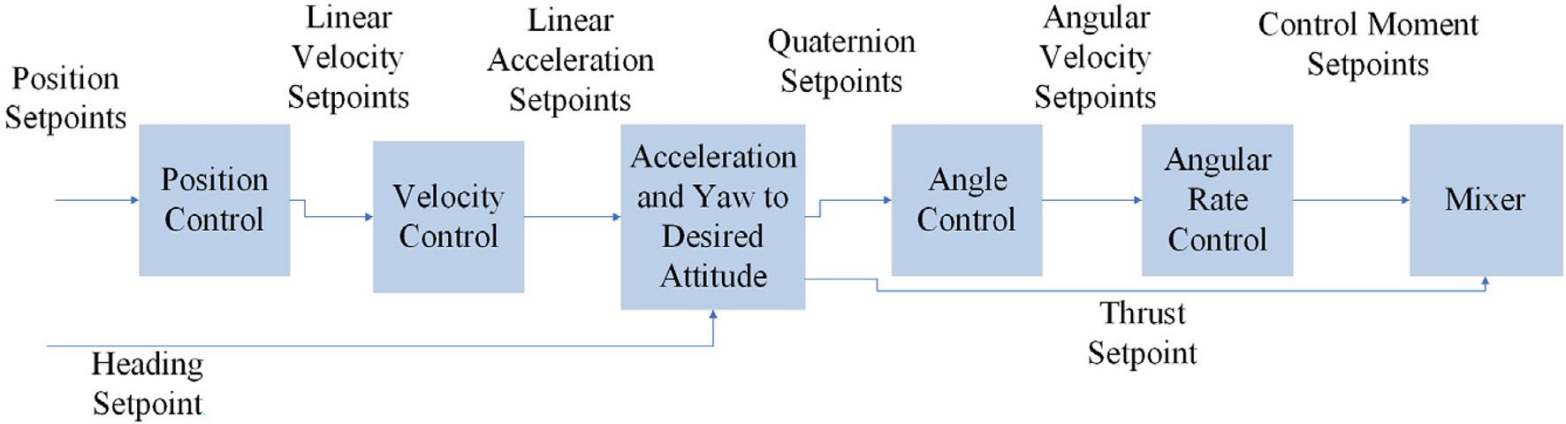
PX4 overall control architecture^23^.

The PX4 control architecture and algorithms can be found in its online document^38^ and source code^59^. The controller structure here is based on^37^, in which a modified and simpler version of the PX4 controller is developed. The overall control architecture of the PX4 autopilot is illustrated in Fig. 30. The controller is a cascade PID with a linear position control loop as the most outer and the angular rate control loop as the most inner control loop. The controller's output is the desired thrust and control moments, which will then be used in the mixer to calculate the rotation speeds for each propeller.

Four control loops should be designed: the linear position control loop, the linear velocity control loop, the angle control loop, and the angular velocity control loop. In each control loop, a PID controller is utilized as:

25 $$SP = K_{p} e + K_{I} \smallint e + K_{d} \dot{e} .{ }$$

26 $$e = x_{sp} - x .{ }$$

In these equations, $x$ and ${x}_{sp}$ are the current and desired value for the state(s) which is meant to be controlled in the corresponding control loop and the SP is the output of the control loop, which then will be inserted to next control loop as desired state. In the position control loop and angle control loop, because the governed equations are kinematic, there is no uncertainty and dynamic behavior; therefore, only the “P” part of the PID controller might be used.

The mixer's “control allocation matrix” should translate the desired thrust and control moments to the motor's PWM (or propeller's rotational speed). The control allocation matrix is determined as follows:

For coaxial propellers which should generate 80% of the total thrust and also control the yaw moment of the multirotor (i.e., the $M_{{z_{B} T}}$)

27 $$\user2{M}_{{\user2{Alloc}_{{\user2{coaxial}}} }} = \left[ {\begin{array}{*{20}c} {C_{{T_{{\omega _{u} }} }} } & {C_{{T_{{\omega _{l} }} }} } \\ {C_{{M_{{\omega _{u} }} }} } & { - C_{{M_{{\omega _{l} }} }} } \\ \end{array} } \right].{\text{~}}$$

28 $$\left[ {\begin{array}{*{20}c} {0.8~T} \\ {M_{{z_{{B_{T} }} }} } \\ \end{array} } \right] = \left[ {\begin{array}{*{20}c} {T_{{coaxial}} } \\ {M_{{z_{B} T_{{coaxial}} }} } \\ \end{array} } \right] = \left[ {\begin{array}{*{20}c} {C_{{T_{{\omega _{u} }} }} } & {C_{{T_{{\omega _{l} }} }} } \\ {C_{{M_{{\omega _{u} }} }} } & { - C_{{M_{{\omega _{l} }} }} } \\ \end{array} } \right]\left[ {\begin{array}{*{20}c} {\omega _{u}^{2} } \\ {\omega _{l}^{2} } \\ \end{array} } \right].~$$

For control propellers which should generate 20% of the total thrust and also control the roll and pitch moments (i.e., $M_{{x_{B} T}}$ and $M_{{y_{B} T}}$). The desired yaw moment of control propellers was set to zero.

29 $${{{\varvec{M}}}_{{\varvec{A}}{\varvec{l}}{\varvec{l}}{\varvec{o}}{\varvec{c}}}}_{{\varvec{c}}{\varvec{o}}{\varvec{a}}{\varvec{x}}{\varvec{i}}{\varvec{a}}{\varvec{l}}}=\left[\begin{array}{cccc}{C}_{{T}_{{\omega }_{1}}}& {C}_{{T}_{{\omega }_{2}}}& {C}_{{T}_{{\omega }_{3}}}& {C}_{{T}_{{\omega }_{4}}}\\ {-L}_{control} {C}_{{T}_{{\omega }_{1}}}& {L}_{control} {C}_{{T}_{{\omega }_{2}}}& {L}_{control} {C}_{{T}_{{\omega }_{3}}}& -{L}_{control} {C}_{{T}_{{\omega }_{4}}}\\ {L}_{control} {C}_{{T}_{{\omega }_{1}}}& {-L}_{control} {C}_{{T}_{{\omega }_{2}}}& {L}_{control} {C}_{{T}_{{\omega }_{3}}}& -{L}_{control} {C}_{{T}_{{\omega }_{4}}}\\ {C}_{{M}_{{\omega }_{1}}}& {C}_{{M}_{{\omega }_{2}}}& {-C}_{{M}_{{\omega }_{3}}}& -{C}_{{M}_{{\omega }_{4}}}\end{array}\right].$$

30 $$\left[\begin{array}{c}\begin{array}{c}{0.2 T}_{total}\\ {{M}_{{x}_{B}}}_{T}\end{array}\\ \begin{array}{c}{{M}_{{y}_{B}}}_{T}\\ 0\end{array}\end{array}\right]=\left[\begin{array}{c}\begin{array}{c}{T}_{control}\\ {{{M}_{{x}_{B}}}_{T}}_{control}\end{array}\\ \begin{array}{c}{{{M}_{{y}_{B}}}_{T}}_{control}\\ {{{M}_{{z}_{B}}}_{T}}_{control}\end{array}\end{array}\right]=\left[\begin{array}{cccc}{C}_{{T}_{{\omega }_{1}}}& {C}_{{T}_{{\omega }_{2}}}& {C}_{{T}_{{\omega }_{3}}}& {C}_{{T}_{{\omega }_{4}}}\\ {-L}_{control} {C}_{{T}_{{\omega }_{1}}}& {C}_{{T}_{{\omega }_{1}}} {C}_{{T}_{{\omega }_{2}}}& {L}_{control} {C}_{{T}_{{\omega }_{3}}}& -{L}_{control} {C}_{{T}_{{\omega }_{4}}}\\ {L}_{control} {C}_{{T}_{{\omega }_{1}}}& {-L}_{control} {C}_{{T}_{{\omega }_{2}}}& {L}_{control} {C}_{{T}_{{\omega }_{3}}}& -{L}_{control} {C}_{{T}_{{\omega }_{4}}}\\ {C}_{{M}_{{\omega }_{1}}}& {C}_{{M}_{{\omega }_{2}}}& {-C}_{{M}_{{\omega }_{3}}}& -{C}_{{M}_{{\omega }_{4}}}\end{array}\right]\left[\begin{array}{c}{\omega }_{1}^{2}\\ \begin{array}{c}{\omega }_{2}^{2}\\ \begin{array}{c}{\omega }_{3}^{2}\\ {\omega }_{4}^{2}\end{array}\end{array}\end{array}\right].$$

In above equations, $C_{{T_{{\omega_{x} }} }}$ and $C_{{M_{{\omega_{x} }} }}$ are thrust coefficient and yaw moment coefficient of the x-th propeller, which can be calculated using Eqs. (31, 32). $L_{control}$ is the distance of each control propeller to the center of gravity in each direction of xy plane of body coordinate system.

31 $$C_{{T_{\omega } }} = \frac{T}{{\omega^{2} }}.{ }$$

32 $$C_{{M_{\omega } }} = \frac{{M_{{z_{B} }} }}{{\omega^{2} }}.{ }$$

#### Simulation

The 6DOF model and the designed control loops were developed in previous sections. In this section, at first, the PID controller coefficients should be tuned to achieve acceptable stability, zero stead y-state error, etc. The tuning process was performed with an initial coefficient of a normal X quadrotor from the PX4 autopilot, changing these coefficients to achieve the desired performance. After tuning was completed. Different scenarios were tested to examine the controller and the multirotor performance. The results for two of these scenarios will be presented here.

**Figure 31:**
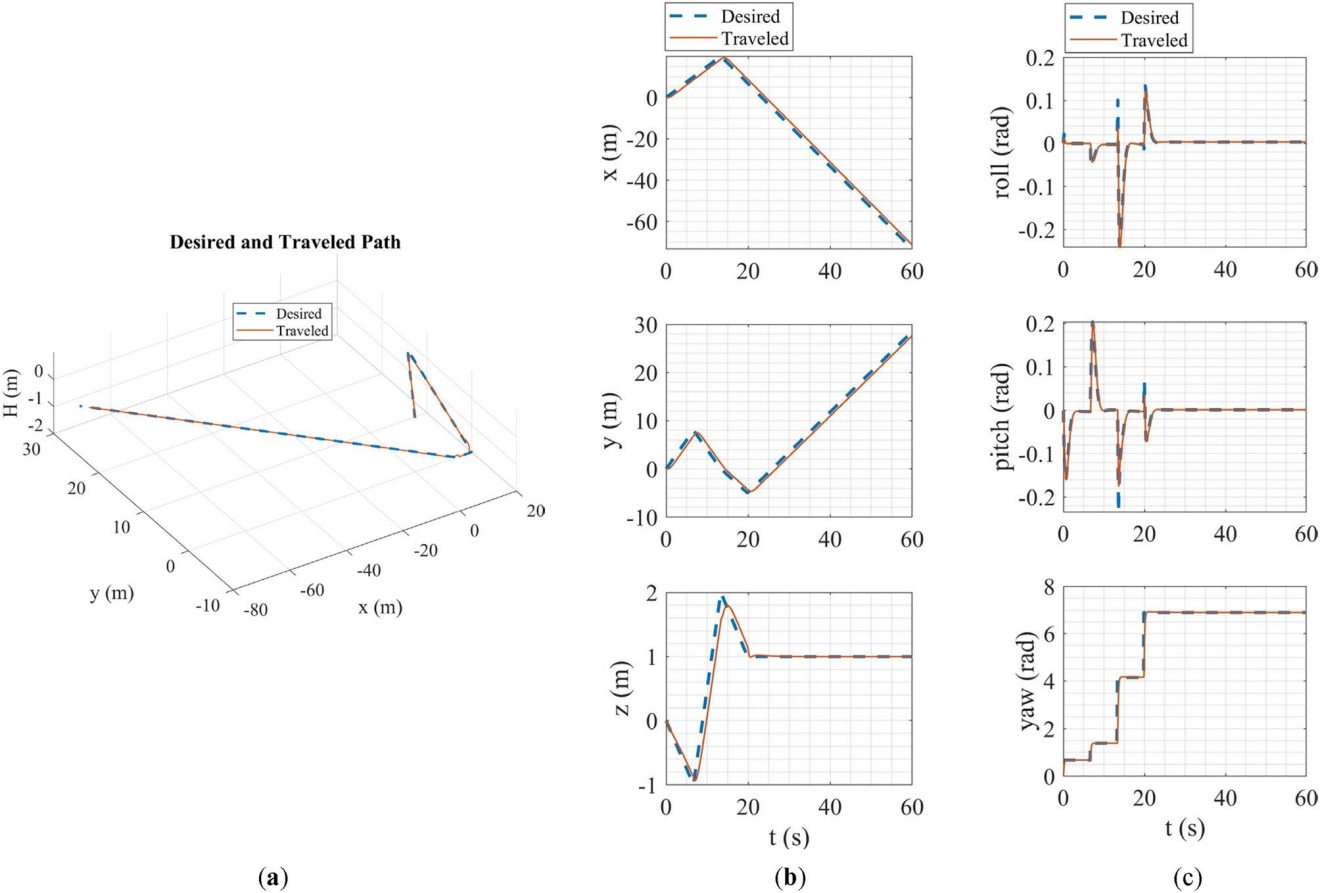
Results of the first scenario in flight dynamic simulation: (**a**) tracking of 3D-trajectory; (**b**) tracking of desired trajectories in each axis of the inertial coordinate system; (**c**) tracking of Euler angles.

**Figure 32:**
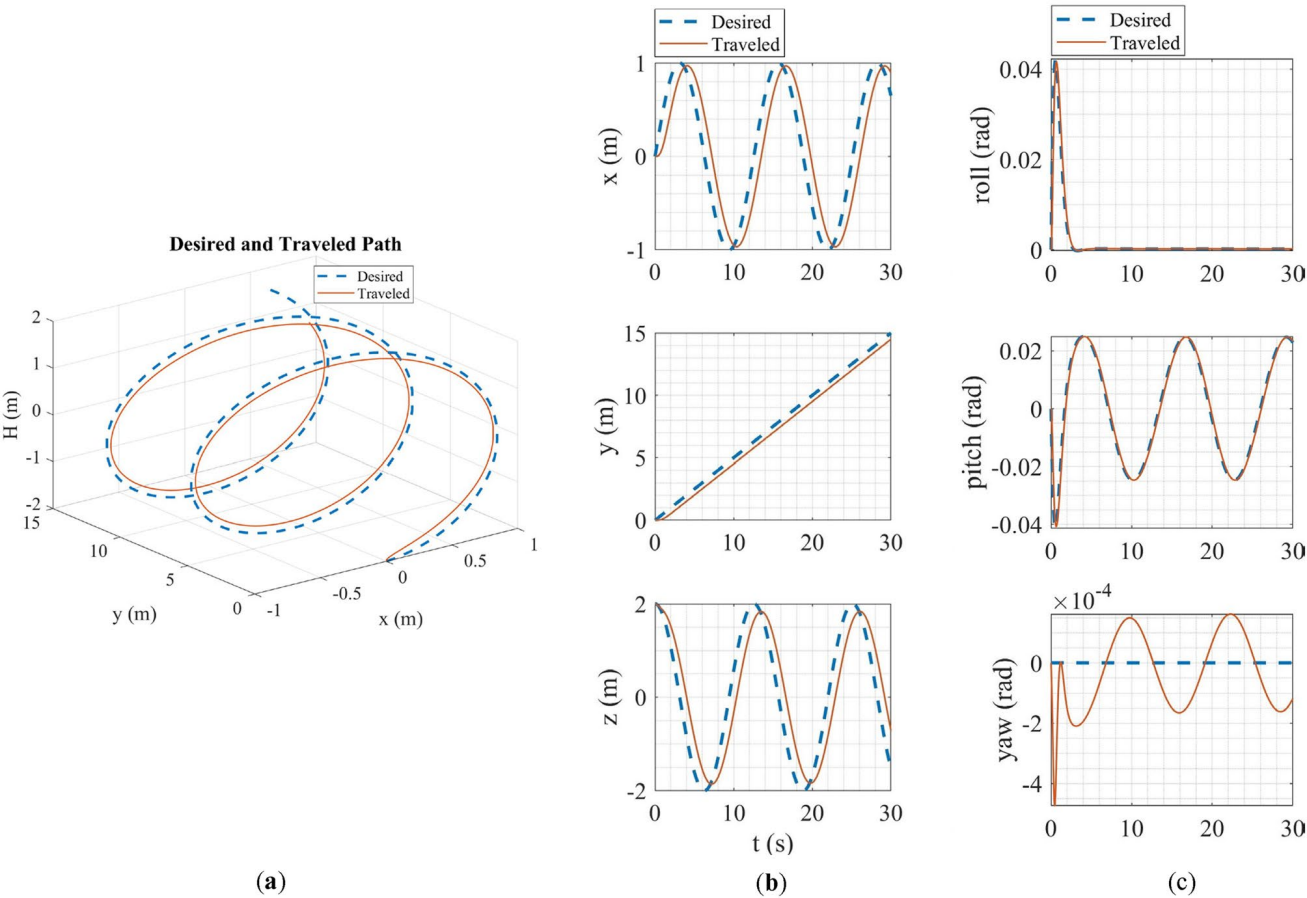
Results of the second scenario in flight dynamic simulation: (**a**) tracking of 3D-trajectory; (**b**) tracking of desired trajectories in each axis of the inertial coordinate system; (**c**) tracking of Euler angles.

- Waypoint tracking: In this scenario, five different points were selected, and the desired trajectory was given to the multirotor as straight lines between each two points. The multirotor should also perform a yaw motion at each set point to be faced toward the next set point. In this scenario, the RMS error for trajectory tracking was 1.8 m, and tracking linear and angular states was acceptable. The results are illustrated in Fig. 31.
- Maneuver flight: In this scenario, a more challenging flight trajectory was given to the multirotor as the desired trajectory. It was a combination of sinusoidal movement in the x and z direction of the earth frame and linear motion in the y direction. The desired yaw value was also set to zero. In this scenario, the RMS error for trajectory tracking was 0.9 m, and tracking linear and angular states was acceptable. The results are illustrated in Fig. 32.

In linear position tracking in all simulations, the maximum steady state error is about 5% with the maximum settling time of 1.2 s and no overshoot. In attitude channels, the maximum steady state error is about 1% with the settling time of 1.1 s and a maximum of 1.2% overshoot.

### Structural analysis

In this stage, the goal is to analyze the multirotor structure in different loading conditions to evaluate its strength and modify the structural design (i.e., material, dimensions, etc.) if necessary. The analysis was performed with two different scenarios, static and dynamic. In static analysis, the multirotor is fixed to the ground, all motors generate their maximum thrust force, and the multirotor weight is considered. In the dynamic analysis, the hard-landing scenario was examined with the multirotor having the initial descent speed of 3 m/s and speed of 1 m/s in each direction of the horizontal plane.

To define the mechanical property of each part, the woven carbon-epoxy composite material properties were imported based on^64–66^; also, the properties of PLA for 3D-print parts were imported based on^67,68^. The necessary interactions and contact properties were selected, and load conditions based on the analyzed scenario were created. The structured mesh was generated with a focus on prioritizing mesh quality. To evaluate the structural strength, for 3D-printed parts, the Von-Mises tension is compared with PLA yield strength considering the safety factor of 1.75^4^. For carbon-epoxy composite parts, different criteria are considered, such as matrix tensile criteria or fiber compression criteria^69^; these criteria will have a value between zero to one; zero means no damage and one means a complete failure. The aim is for all composite parts to have a value of zero in all damage measures.

#### Static analysis

**Figure 33:**
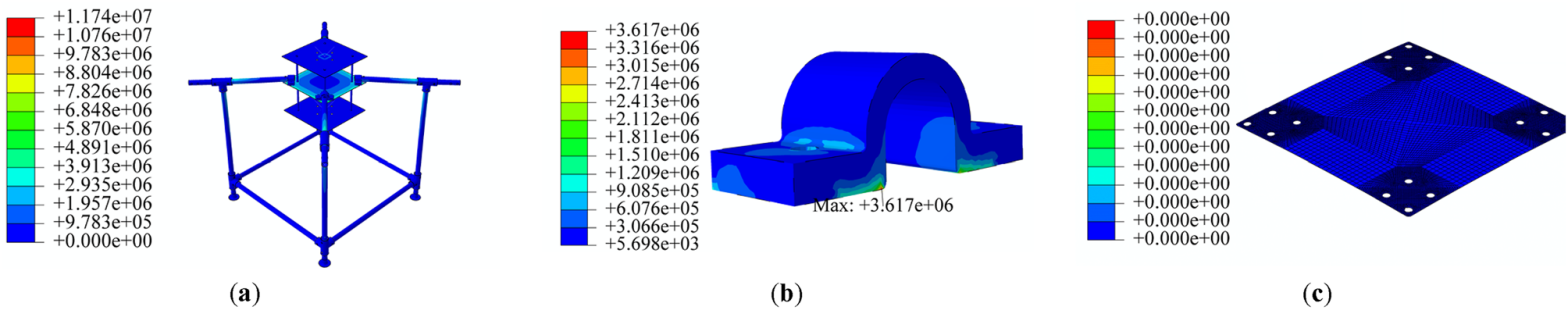
Multirotor under static load condition: (**a**) structure tension and deformation; (**b**) arm-to-main-plate connector Von-Mises tension; (**c**) the shear damage criteria for middle plate.

The multirotor landing skids were fixed, and the maximum thrust force of each motor was applied into each motor installation point. The mass of the multirotor and the gravity acceleration were also considered. The Von-Mises tension on the multirotor structure in this loading condition is illustrated in Fig. 33a. Among all 3D-printed parts, the arm-to-main-plate connector is under the maximum tension of about 4 MPa, as shown in Fig. 33b, which is acceptable considering the tensile strength of PLA (70 MPa). Between composite parts, the middle plate is under the maximum tension, but all damage criteria for this plate are equal to zero. The shear damage criteria are presented here as an example in Fig. 33c.

#### Dynamic analysis

**Figure 34:**
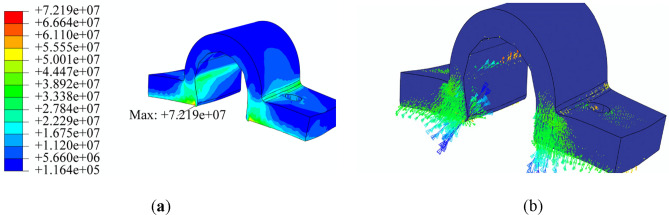
More detailed investigation of arm-to-main-plate-connector tension.

A rectangular rigid body that represents the landing area was added to the analysis. The multirotor was initialized with a 3 m/s speed downward and 1 m/s speed in each direction of the horizontal plane. The mass of the multirotor and gravity acceleration were also considered. At each impact step, different composite parts experienced various ranges of tension conditions. Therefore, it was necessary to investigate the damage criteria for all of them in all steps of landing and impact. All composite parts have a value of zero in each damage criterion. Among all 3D-printed parts, the arm-to-main-plate connector is under maximum tension again. The maximum stress at some landing steps is more than PLA yield strength. As illustrated in Fig. 34, this tension is mainly in the form of compression, which will not cause the failure, but it’s necessary to check this part after a hard-landing occurrence.

## System analysis and validation

In this section, the flight test results of the multirotor are presented. As mentioned in section “Overall design and development procedure”, flight tests in this section aim to evaluate the multirotor functionality as a system and analyze its overall performance. Three flight modes were tested in flight tests: stabilized flight mode, altitude flight mode and Auto flight mode.

- Stabilized Flight mode

**Figure 35:**
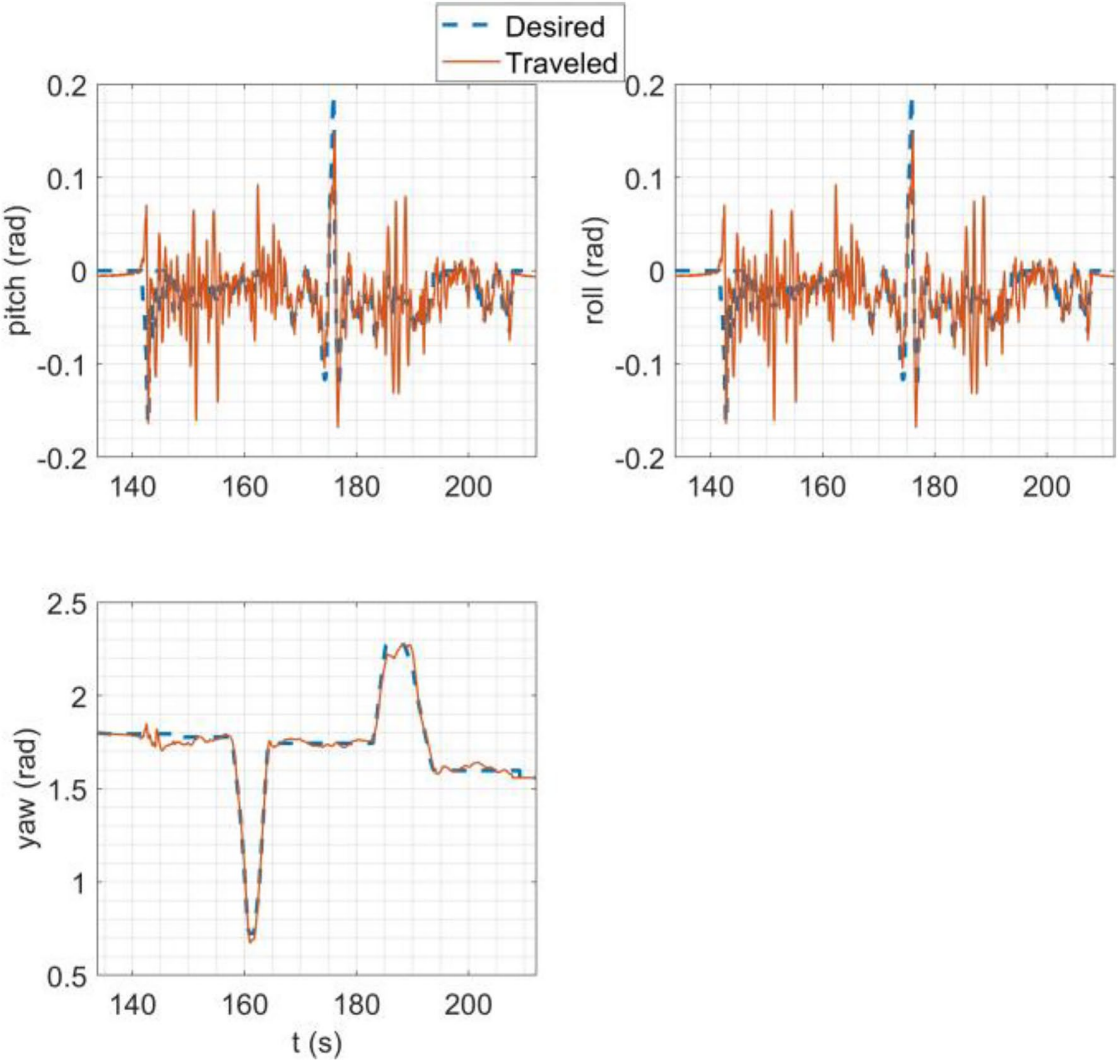
Tracking of desired Euler angles in flight test in stabilize flight mode.

Tracking of desired Euler angles is illustrated in Fig. 35.

The multirotor experienced a significant ground effect in takeoff and landing phase, but the multirotor was able to hold its attitude correctly and handling quality and response to the pilot inputs were acceptable. The flight test video and the flight log can be found as supplementary video S1 and supplementary log L1 online, respectively.

- Altitude Flight mode

**Figure 36:**
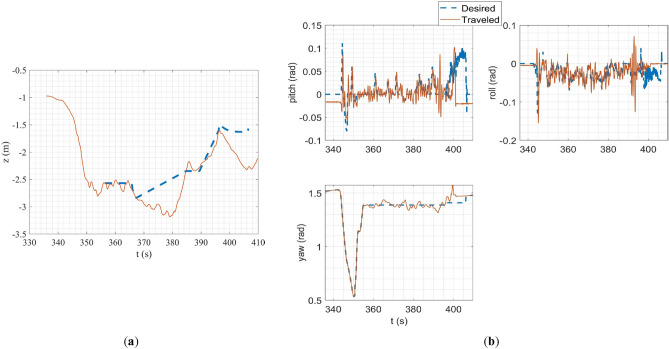
Altitude mode flight test result: (**a**) tracking of euler angles; (**b**) tracking of desired position in z-direction trajectory.

Tracking of desired Euler angles and desired altitude are illustrated in Fig. 36. The multirotor was able to hold its altitude correctly and handling quality and response to the pilot inputs were acceptable. The flight test video and the flight log can be found as supplementary video S2 and supplementary log L2 online, respectively.

- Auto Flight mode

**Figure 37:**
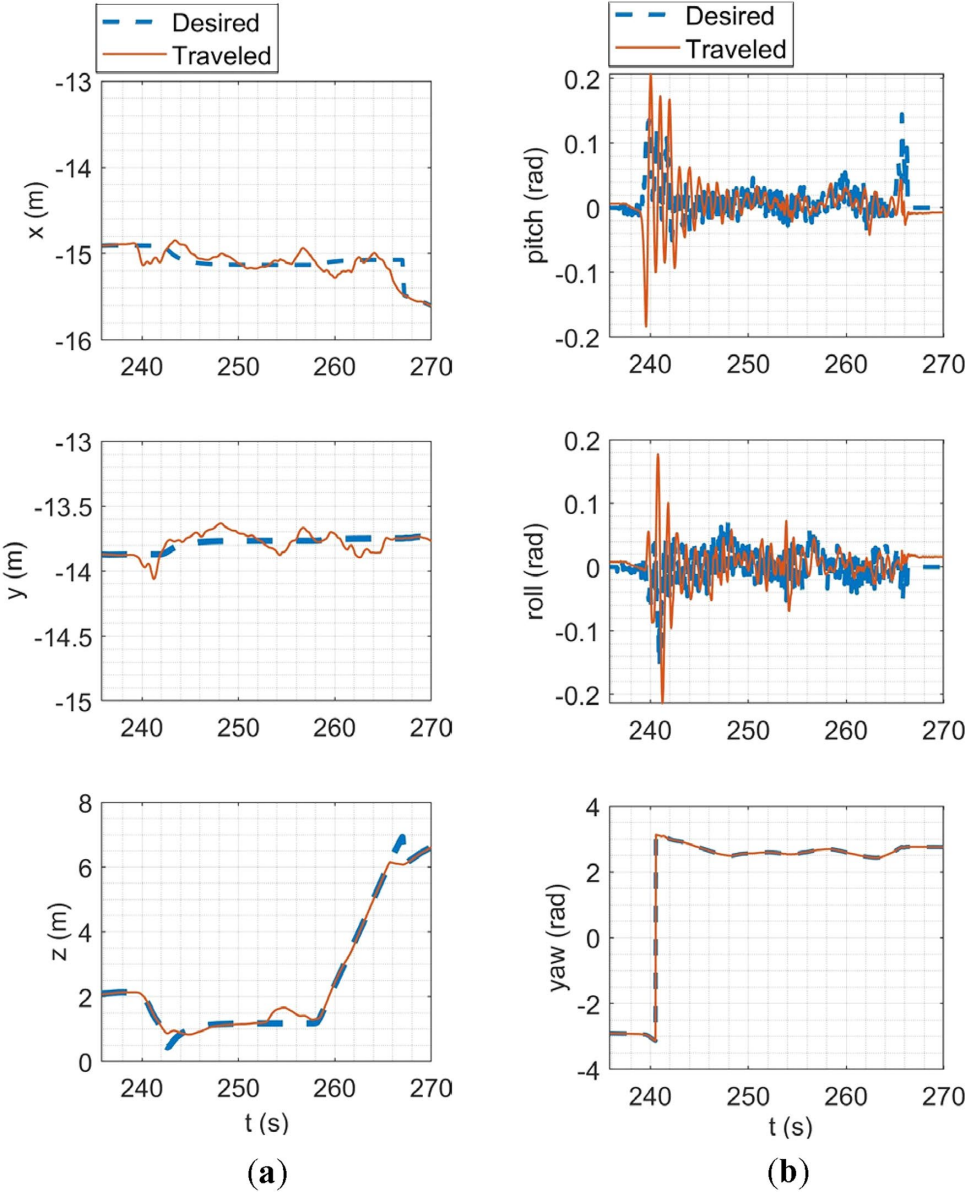
Flight test Auto mode: (**a**) tracking of desired trajectories in each axis of the inertial coordinate system; (**b**) tracking of Euler angles.

In this test, the multirotor performed an auto takeoff, then had a hover flight in which it hold its position and altitude for about 10 s and after that, it landed automatically. Tracking of desired Euler angles and 3D position is illustrated in Fig. 37. The flight test video and the flight log can be found as supplementary video S3 and supplementary log L3 online, respectively.

Overall flight performance of the multirotor was acceptable, although some oscillation was obvious when the multirotor was close to the ground due to the ground defect of the lower propeller. In linear position tracking in auto mode, the maximum steady state error is about 2% with the maximum settling time of 2 s and no overshoot. In attitude channels, the maximum steady state error is about 1% with the settling time of 1.1 s and a maximum of 2% overshoot.

## Conclusion and future works

In this study, the design and development of a multirotor with a novel configuration were presented to provide proof of concept of this novel configuration and establish a systematic design and development procedure based on the V-method approach. The design was carried out at different levels, from the system level to detailed components design, and in each stage, a proper validation process was presented.

While the results from various validation stages confirm the functionality of the multirotor and its significance for future research, they also reveal certain disadvantages and limitations associated with both the configuration and the development process.

- One potential disadvantage of the novel configuration, as highlighted in the aerodynamic analysis section, is the limitation on maximum climb speed. This limitation arises from the increase in axial flow on the inner part of the lower propeller, leading to a reduction in its thrust and yaw moment, potentially causing instability issues. Addressing this challenge may involve utilizing various optimization methods to enhance the performance of the coaxial system.
- Utilizing coaxial propellers at the center of the configuration introduces various mechanical complexities. These challenges encompass the installation and assembly of the coaxial system (such as design three outlined in section “Overall configuration of the multirotor”) and the placement of other components around the propellers. It is crucial to thoroughly address this issue in further development, particularly for hybrid configurations.
- The incorporation of coaxial propellers also complicates payload placement. Positioning the payload under the coaxial system can introduce various challenges, including reduced efficiency and increased ground effect on the lower propeller.
- An important observation from flight tests was the multirotor's oscillation in close proximity to the ground, attributed to ground effects on the lower propeller. This phenomenon can worsen when the payload is installed under the coaxial system. While the multirotor successfully performed takeoff and landing in both manual and auto flight modes, this limitation should be carefully addressed in future studies.
- Utilizing different optimization methods to improve the design and development procedure presented in the current study. In different stages of design, a wide range of decisions need to be made; using optimization methods, these decisions can be made faster, be more accurate, and consider a vast range of possible options.
- For commercial development purposes, more disciplines should be considered in the design and development process, incorporating various factors such as cost, maintenance, weight, flight performance, and other interrelated aspects.
- Improve each validation stage using more accurate analysis tools like a wind tunnel, drop test facility, etc.
- Proper hardware and software tools are needed to evaluate multirotor performance characteristics like those developed in^70^. Using these tools, the performance of the developed multirotor in the current study can be compared with conventional configuration in a safer, more rapid, and more accurate manner.

### Supplementary Information

Supplementary Information.

## Data Availability

All data generated or analysed during this study are included in this published article and supplementary data.

## References

[1] Delbecq S, Budinger M, Ochotorena A, Reysset A, Defaÿ F (2020). Efficient sizing and optimization of multirotor drones based on scaling laws and similarity models. Aerosp. Sci. Technol..

[2] Magnussen Ø, Ottestad M, Hovland G (2015). Multicopter Design Optimization and Validation.

[3] Klimczyk WA (2022). Aerodynamic design and optimization of propellers for multirotor. Aircr. Eng. Aerosp. Technol..

[4] Kumar, V. A. *et al*. Structural optimization of frame of the multi-rotor unmanned aerial vehicle through computational structural analysis. In *Proceedings of the Journal of Physics: Conference Series* 012004 (2021).

[5] Jarrah K, Alali Y, Lalko A, Rawashdeh O (2022). Flight time optimization and modeling of a hybrid gasoline–electric multirotor drone: An experimental study. Aerospace.

[6] Dominguez VH, Garcia-Salazar O, Amezquita-Brooks L, Reyes-Osorio LA, Santana-Delgado C, Rojo-Rodriguez EG (2022). Micro coaxial drone: Fflight dynamics, simulation and ground testing. Aerospace.

[7] Wei Y, Chen H, Li K, Deng H, Li D (2019). Research on the control algorithm of coaxial rotor aircraft based on sliding mode and PID. Electronics.

[8] Mathur, A. & Atkins, E. M. Design, modeling and hybrid control of a quadplane. In *Proceedings of the AIAA Scitech 2021 Forum* 0374 (2021).

[9] McCormick BW (1994). Aerodynamics, Aeronautics and Flight Mechanics.

[10] Maisel, M. D. *The History of the XV-15 Tilt Rotor Research Aircraft: From Concept to Flight* (National Aeronautics and Space Administration, Office of Policy and Plans, 2000).

[11] Merchant, M. P. *Propeller Performance Measurement for Low Reynolds Number Unmanned Aerial Vehicle Applications* (Wichita State University, 2005).

[12] Brandt, J. & Selig, M. Propeller performance data at low reynolds numbers. In *Proceedings of the 49th AIAA Aerospace Sciences Meeting including the New Horizons Forum and Aerospace Exposition* 1255 (2011).

[13] Theys, B., Dimitriadis, G., Hendrick, P. & De Schutter, J. Influence of propeller configuration on propulsion system efficiency of multi-rotor Unmanned Aerial Vehicles. In *Proceedings of the 2016 International Conference on Unmanned Aircraft Systems (ICUAS) *195–201 (2016).

[14] Saadat S, Esmailifar SM, Masroor F (2022). Design of a hybrid heavy multirotor with long range and high payload carrying capacity for using in disaster management. Aerosp. Knowl. Technol. J..

[15] Russian unmanned, long-range, hybrid multirotor wants to do the heavy lifting. (2023, accessed 2024) https://newatlas.com/ardn-russia-skyf-heavy-lift-hybrid-multirotor/52300/.

[16] Piancastelli L, Sali M (2023). Tri-rotor propeller design concept, optimization and analysis of the lift efficiency during hovering. Arab. J. Sci. Eng..

[17] Amado, I. S. Experimental comparison of planar and coaxial rotor configurations in multi-rotors. In *Instituto Superior Técnico* (2017).

[18] Leishman JG, Ananthan S (2008). An optimum coaxial rotor system for axial flight. J. Am. Helicopter Soc..

[19] Duan, X. Multidisciplinary design optimization of coaxial drone propellers. In *University of Toronto (Canada)* (2022).

[20] Günther AOF (2020). Co-axial Propeller Configuration Optimization.

[21] ASCEND AEROSYSTEM. (2023) https://ascentaerosystems.com/spirit/.

[22] DJI. (2023, accessed 2023) https://www.dji.com/.

[23] AUTEL ROBOTICS. https://www.autelrobotics.com/.

[24] Roskam, J. *Airplane Design* (DARcorporation, 1985).

[25] Quan Q (2017). Introduction to Multicopter Design and Control.

[26] de Angelis EL, Giulietti F, Rossetti G, Bellani G (2021). Performance analysis and optimal sizing of electric multirotors. Aerosp. Sci. Technol..

[27] Goli S, Kurtuluş DF, Alhems LM, Memon AM, Imran IH (2023). Experimental study on efficient propulsion system for multicopter UAV design applications. Results Eng..

[28] Månsson C, Stenberg D (2014). Model-Based Design Development and Control of a Wind Resistant Multirotor UAV.

[29] Tsadok T (2014). Thruster Modeling for Small Unmanned Aerial Vehicles with Coaxial-Rotors.

[30] Shamsudin SS, Madzni MZ (2021). Aerodynamic analysis of quadrotor uav propeller using computational fluid dynamic. J. Complex Flow.

[31] Kutty HA, Rajendran P (2017). 3D CFD simulation and experimental validation of small APC slow flyer propeller blade. Aerospace.

[32] Gill, R. & D'andrea, R. Propeller thrust and drag in forward flight. In *Proceedings of the 2017 IEEE Conference on control technology and applications (CCTA)* 73–79 (2017).

[33] Bangura, M. Aerodynamics and control of quadrotors. In *The Australian National University (Australia)* (2017).

[34] Hattenberger G, Bronz M, Condomines J-P (2023). Evaluation of drag coefficient for a quadrotor model. Int. J. Micro Air Veh..

[35] Almallah S, Elnady A (2022). CFD Analysis of Full Quadcopter.

[36] Weerasinghe S, Monasor M (2017). Simulation and Experimental Analysis of Hovering and Flight of a Quadrotor.

[37] Quan Q, Dai X, Wang S (2020). Multicopter Design and Control Practice: A Series Experiments Based on MATLAB and Pixhawk.

[38] PX4 Autopilot: Open Source Autopilot for Drones (2023, accessed 2023). https://px4.io/.

[39] Zhih, C. C., Ragavan, S. K. V. & Shanmugavel, M. Development of a simple, low-cost autopilot system for multi-rotor UAVs. In *Proceedings of the 2015 IEEE Recent Advances in Intelligent Computational Systems (RAICS)* 285–289 (2015).

[40] Khrenov, A. V. & Diane, S. A. Multi-module quadcopter with autopilot based on raspberry PI. In *Proceedings of the 2021 IEEE Conference of Russian Young Researchers in Electrical and Electronic Engineering (ElConRus)* 2118–2123 (2021).

[41] Carvalho, J. P. *et al*. Autonomous UAV outdoor flight controlled by an embedded system using Odroid and ROS. In *Proceedings of the CONTROLO 2016: Proceedings of the 12th Portuguese Conference on Automatic Control* 423–437 (2017).

[42] UAV toolbox support package for PX4 autopilots (2023, accessed 2023). https://www.mathworks.com/help/supportpkg/px4/.

[43] Goel, A., Salim, A. M., Ansari, A., Ravela, S. & Bernstein, D. Adaptive digital pid control of a quadcopter with unknown dynamics. arXiv:2006.00416 (2020).

[44] Nguyen, K. D.,Ha, C., Jang, J. T. Development of a new hybrid drone and software-in-the-loop simulation using px4 code. In *Proceedings of the Intelligent Computing Theories and Application: 14th International Conference, ICIC 2018, Wuhan, China, August 15–18, 2018, Proceedings, Part I 14* 84–93 (2018).

[45] Singh R, Kumar R, Mishra A, Agarwal A (2020). Structural analysis of quadcopter frame. Mater. Today: Proc.

[46] Raymer D (2012). Aircraft Design: A Conceptual Approach.

[47] Martlet MI-2 tactical UAS | long range ISR drone (2023, accessed 2023). https://heighttechnologies.com/products/mi-1/.

[48] Yuneec (2023, accessed 2023). https://yuneec.online/.

[49] SwellPro Official Site | Waterproof Drone Pioneer (2023, accessed 2023). https://swellpro-uk.co.uk/.

[50] Tarot 650 v2.2 Ready To Fly Drone—UAV Systems International (2023, accessed 2023). https://uavsystemsinternational.com/products/tarot-650-ready-to-fly-drone.

[51] T-motor (2023, accessed 2023). https://store.tmotor.com/.

[52] Biczyski M, Sehab R, Whidborne JF, Krebs G, Luk P (2020). Multirotor sizing methodology with flight time estimation. J. Adv. Transport..

[53] Xiao, A., Park, S. S., Freiheit, T. A comparison of concept selection in concept scoring and axiomatic design methods. In *Proceedings of the Canadian Engineering Education Association (CEEA)* (2007).

[54] Mohebbi A, Achiche S, Baron L (2018). Multi-criteria fuzzy decision support for conceptual evaluation in design of mechatronic systems: A quadrotor design case study. Res. Eng. Design.

[55] Cai, J. *Changes in Propeller Performance Due to Ground and Partial Ground Proximity* (University of Dayton, 2020).

[56] iFlight Carbon Fiber Props 12 x 4.5 APC Style (2023, accessed 2023). https://www.radiocontrolledshop.ie/multirotor-props-multicopter-propellers-quadcopter-props-racing-quad-propellers/10925-iflight-carbon-fiber-props-12-x-45-apc-style-icf-1245-dublin-ireland.html.

[57] Hobbypower 30a Brushless Speed Controller ESC for X525 Multicopter Helicopter Airplane (2023, accessed 2023). https://www.amazon.com/Hobbypower-Brushless-Controller-Multicopter-Helicopter/dp/B00XKX5TBE?th=1.

[58] HobbyKing 10A (2~3S) ESC 1A UBEC (2023, accessed 2023). https://hobbyking.com/en_us/hobbyking-10a-2-3s-esc-1a-ubec.html.

[59] PX4 Autopilot Software (2023, accessed 2023). https://github.com/PX4/PX4-Autopilot.

[60] Gazebo (2023, accessed 2023). https://gazebosim.org/.

[61] QGC—QgroundControl—Drone Control (2023, accessed 2023). http://qgroundcontrol.com/.

[62] White FM (1966). Fluid Mechanics.

[63] Zipfel PH (2000). Modeling and Simulation of Aerospace Vehicle Dynamics.

[64] Feito Sánchez, N., Díaz Álvarez, A., Cantero Guisández, J. L., Rodríguez Millán, M. & Miguélez Garrido, M. H. *Experimental Analysis of Special Tool Geometries when Drilling Woven and Multidirectional CFRPs* (2016).

[65] Ahmad H, Crocombe A, Smith P (2013). Physically based finite element strength prediction in notched woven laminates under quasi-static loading. Plast. Rubber Compos..

[66] Riccio A, Saputo S, Sellitto A, Russo A, Di Caprio F, Di Palma L (2019). An Insight on the crashworthiness behavior of a full-scale composite fuselage section at different impact angles. Aerospace.

[67] Ezeh O, Susmel L (2018). On the fatigue strength of 3D-printed polylactide (PLA). Procedia Struct. Integr..

[68] Travieso-Rodriguez JA, Jerez-Mesa R, Llumà J, Traver-Ramos O, Gomez-Gras G, Roa-Rovira JJ (2019). Mechanical properties of 3D-printing polylactic acid parts subjected to bending stress and fatigue testing. Materials.

[69] Kaw AK (2005). Mechanics of Composite Materials.

[70] Bauersfeld L, Scaramuzza D (2022). Range, endurance, and optimal speed estimates for multicopters. IEEE Robot. Autom. Lett..

